# Transarterial Embolization Versus Manipulation Under Anesthesia and Arthroscopic Capsular Release for Refractory Adhesive Capsulitis—A Cost-Effectiveness Analysis

**DOI:** 10.1007/s00270-026-04563-8

**Published:** 2026-08-02

**Authors:** Kameel Khabaz, Rashed Alananzeh, Layth Alkhani, Sammy Allaw, Xiao Wu, Qian Yu, Mikin Patel, Osman Ahmed

**Affiliations:** 1 David Geffen School of Medicine at UCLA, Los Angeles, CA 90095 USA; 2 Joint & Vascular Institute, Libertyville, IL 60048 USA; 3 Northwestern University Feinberg School of Medicine, Chicago, IL 60611 USA; 4 Department of Radiology, University of Chicago, Chicago, IL 60637 USA; 5 Department of Radiology and Biomedical Imaging, University of California San Francisco, San Francisco, CA 94143 USA; 6 Department of Radiology, University of Utah, Salt Lake City, UT 84132 USA

**Keywords:** Musculoskeletal interventions, Embolization, Health system economics, Cost-effectiveness analysis, Systematic review, Meta-analysis, Adhesive capsulitis

## Abstract

This study compared the cost-effectiveness of transarterial embolization (TAE), manipulation under anesthesia (MUA), and arthroscopic capsular release (ACR) for refractory primary adhesive capsulitis. A systematic review with single-arm meta-analyses informed a three-state Markov model over a 36-month horizon. Clinical success, defined as mild pain at 3 months, was highest for TAE (73.3%), followed by ACR (61.4%) and MUA (55.5%). At a $100,000 per quality-adjusted life-year (QALY) threshold, TAE was the optimal strategy (incremental cost-effectiveness ratio vs. ACR: $77,807/QALY). When success was defined by range-of-motion recovery, MUA and ACR yielded more QALYs at lower cost. Based on indirect comparisons, these findings suggest TAE may be a cost-effective option for pain-predominant adhesive capsulitis, while MUA and ACR may be preferred when mechanical restriction is the primary target.

**Graphical Abstract:**

**Supplementary Information:**

The online version contains supplementary material available at https://doi.org/10.1007/s00270-026-04563-8.

**Figure:**
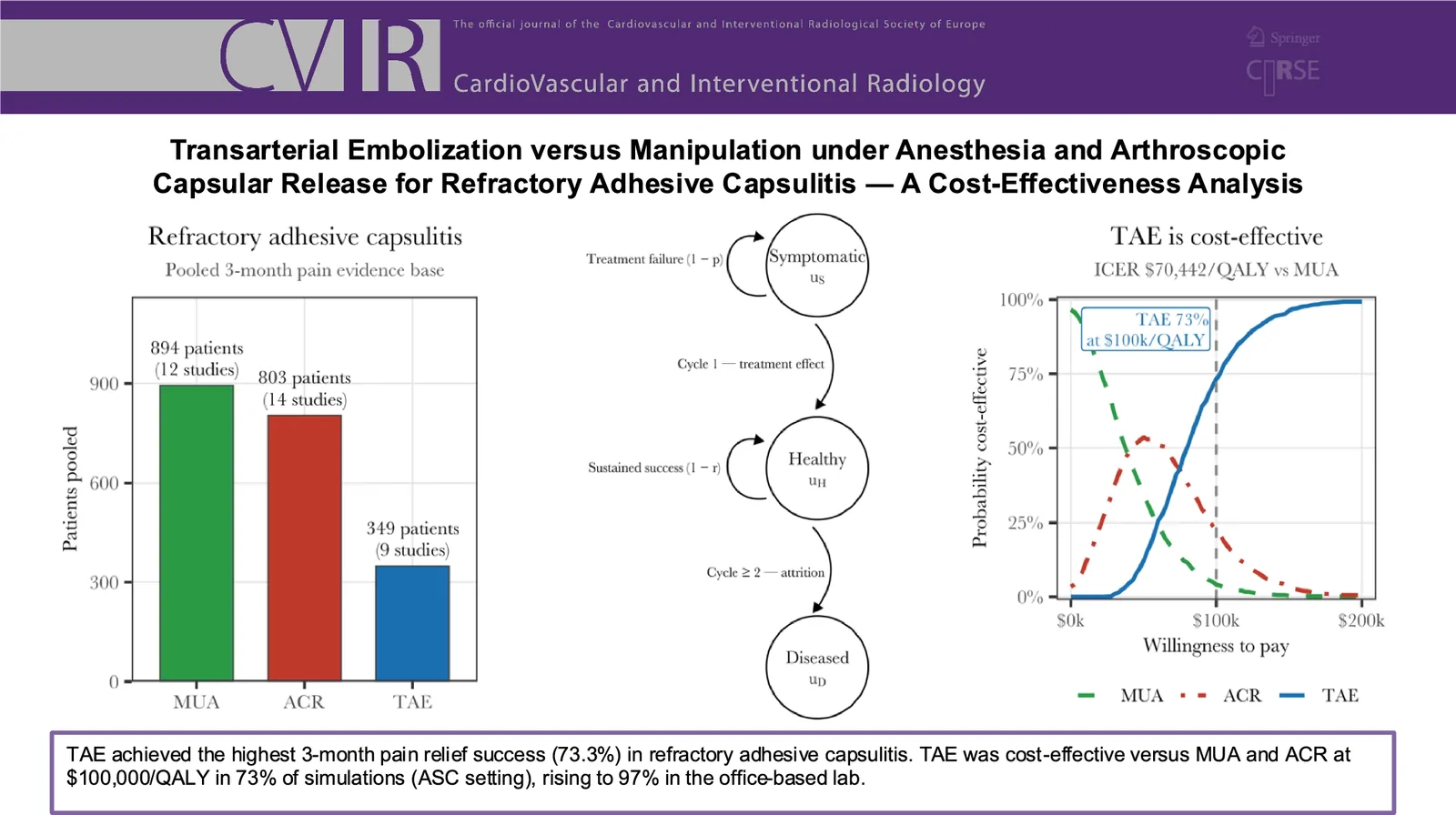

## Introduction

Primary adhesive capsulitis (AC) is the gradual onset of shoulder pain with progressive restriction of glenohumeral motion in the absence of a traumatic, surgical, or neurologic cause [1,2 ]. The condition affects around 2% of the general population, with female predominance and peak incidence after age 40, with risk factors including diabetes, hypothyroidism, and autoimmune diseases [3]. The clinical course progresses through three overlapping phases of pain, stiffness, and recovery [4–6].

While first-line management follows a stepwise conservative approach that includes nonsteroidal anti-inflammatory drugs (NSAIDs), physical therapy, and intra-articular injections, refractory cases require procedural intervention [2,7 ]. Manipulation under anesthesia (MUA) involves controlled stretching and tearing of the capsule under general anesthesia, while arthroscopic capsular release (ACR) entails surgical capsular division under direct visualization. Transarterial embolization (TAE) is a minimally invasive approach entailing selective catheterization and embolization of periarticular neovascularity [8]. Despite established evidence for MUA and ACR and emerging evidence for TAE, no published health economic comparison has integrated the three management approaches.

This study aims to compare the safety, efficacy, and cost-effectiveness of TAE with these alternative management options. Given the mechanistic distinction between the inflammatory and fibrotic drivers of AC, a secondary objective was to evaluate whether cost-effectiveness varies by outcome definition, pain relief versus range-of-motion recovery, to inform treatment selection based on the predominant clinical phenotype.

## Materials and Methods

### Study Design and Reporting Frameworks

We conducted a systematic review with a comparative single-arm meta-analyses and a cost-effectiveness analysis (CEA) comparing TAE, MUA, and ACR for primary AC of the shoulder refractory to conservative management from a U.S. Medicare payer perspective. Reporting follows the Preferred Reporting Items for Systematic Review and Meta-Analyses (PRISMA) 2020 guidelines. The economic evaluation is reported in accordance with the Consolidated Health Economic Evaluation Reporting Standards (CHEERS) 2022. The protocol was registered with PROSPERO (CRD420261370430).

### Eligibility Criteria and Search

**Fig. 1:**
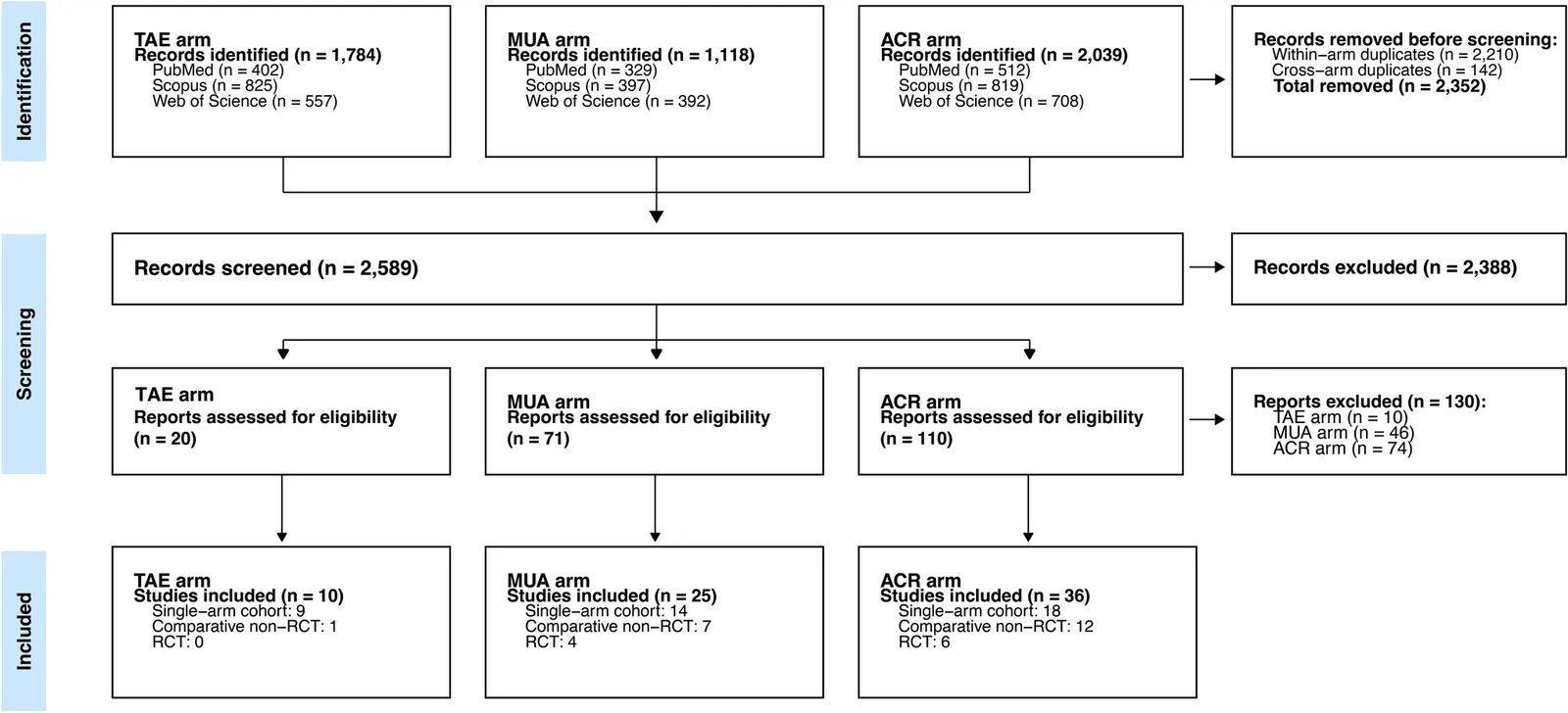
PRISMA flow diagram of included studies. Across 3 databases, 2,589 records were screened across the three arms. After exclusion of 2,388 from primary screening, full-text review was performed on 20 TAE studies, 71 MUA studies, and 110 ACR studies. Nine single-arm and 1 comparative non-RCT study were included in the TAE arm. Fourteen single-arm, 7 comparative non-RCT, and 4 RCT studies were included in the MUA arm. Eighteen single-arm, 12 comparative non-RCT, and 6 RCT studies were included in the ACR arm

Studies on adults with primary adhesive capsulitis who had failed conservative management and underwent TAE, MUA, or ACR were included. PubMed, Scopus, and Web of Science were searched from inception to April 14, 2026 (Fig. 1). Full search strings are in Supplemental Methods S1. Studies were eligible if they enrolled adults with primary AC and reported pre- and post-intervention data for a validated outcome at a mappable time point for greater than 5 shoulders per arm. Studies of secondary AC, combined interventions, and those lacking extractable data were excluded. Risk-of-bias assessment was performed for all studies using the Cochrane Risk-of-Bias 2 (RoB 2) tool for randomized controlled trials (RCTs) and the Risk of Bias in Non-Randomized Studies of Interventions (ROBINS-I) tool for non-randomized studies, with assessments including visualizations, contour-enhanced funnel plots with formal asymmetry testing, and a leave-one-TAE-trial-out sensitivity analysis (see Supplemental Methods S2) [9–11]. Primary screening, full-text review, and data extraction were performed in duplicate (KK, RA) with third reader adjudication (LA).

### Outcomes

The primary clinical outcome was pain (numeric rating scale [NRS] or visual analog scale [VAS], harmonized to 0–10). Secondary outcomes included shoulder function scores, the EQ-5D health utility index, and range-of-motion (ROM) data, including forward elevation, abduction, and external/internal rotation. Serious adverse events (SAE), defined according to the UK Frozen Shoulder Trial (UK FroST), and subsequent-procedure rates, including either repeat intervention or escalation of therapy, were extracted [12]. Outcomes were analyzed at 1, 3, 6, and 12 months. Further detail is provided in Supplemental Methods S3.

### Comparative Meta-Analysis

For each treatment arm, outcome, and timepoint, pre-to-post mean differences (MDs) were pooled using a random-effects fit by restricted maximum likelihood for the between-study variance τ2, with Hartung-Knapp-Sidik-Jonkman confidence intervals [13]. Studies were weighted by the inverse of the sum of their sampling variance and the estimated between-study variance. The I2 statistic, τ2, and the 95% prediction interval were reported for every pool. The 3-month timepoint was chosen as the primary timepoint. Adverse-event and SAE rates were pooled as the aggregate proportion. Statistical significance was set at *p* = 0.05.

### Cost-Effectiveness Analysis

**Fig. 2:**
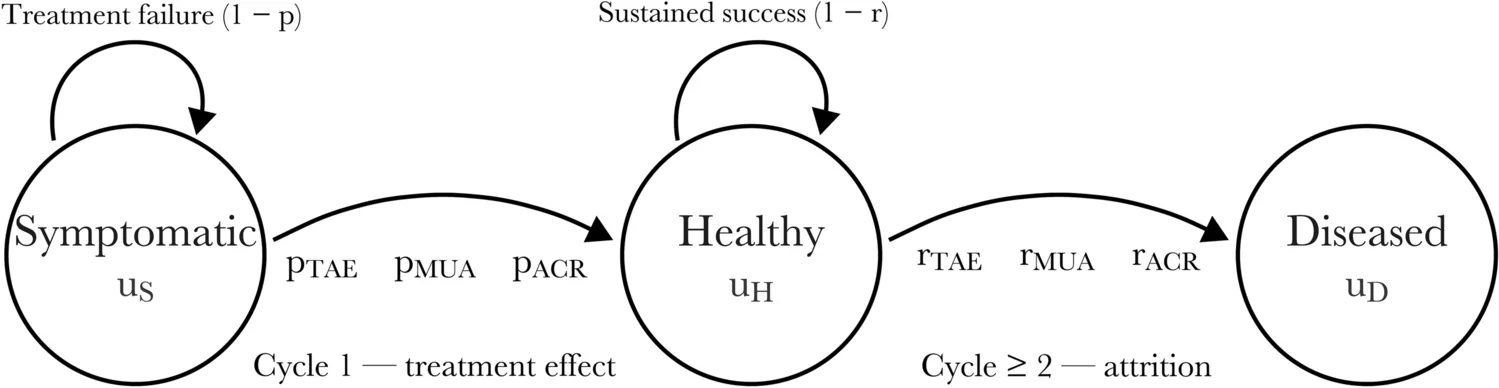
Markov cost-effectiveness model. The three-state Markov model used for CEA consists of a Symptomatic state, at which all simulated patients begin, with a specified transition probability defined by treatment success to the Healthy state after the first cycle. Patients who fail treatment remain in the Symptomatic state. At each cycle thereafter, Healthy patients may either remain in the Healthy state or transition to a refractory Diseased state according to an attrition probability defined by the pooled subsequent-procedure rate. The Healthy state accumulates higher utility scores, shared across arms, than the Symptomatic and Diseased states

A three-state Markov model (Symptomatic, Healthy, Diseased) with quarterly cycles was built from the U.S. Medicare payer perspective over a 36-month horizon following AC’s natural history, with a 3% annual discount rate applied to costs and quality-adjusted life-years (QALYs) [5,14 ]. All patients begin in the Symptomatic state and can reach the Healthy state only by achieving treatment success in cycle 1; those who do not remain Symptomatic, as the cohort is refractory to conservative care with no spontaneous resolution over the time horizon. In later cycles, Healthy patients may transition to the Diseased state at an arm-specific quarterly rate equal to the pooled subsequent-procedure rate (Fig. 2). The Diseased state is absorbing because the included studies did not consistently report the type, outcome, or durability of subsequent procedures, precluding reliable modeling of re-intervention efficacy or cost. Further detail is provided in Supplemental Methods S4.

Clinical success was derived from the single-arm meta-analyses. Each arm’s random-effects pooled 3-month MD in pain was subtracted from a standardized reference baseline of 7, adopted from the UK FroST, to yield the expected post-treatment pain score for that arm [15]. The residual patient-level dispersion was the sample size-weighted mean of reported post-treatment standard deviations, and the transition probability from Symptomatic to Healthy was computed as the proportion of the post-treatment distribution falling at or below a pain NRS of 3. This anchored absolute-threshold formulation was chosen to account for baseline heterogeneity across arms [16]. A secondary analysis defining clinical success according to forward elevation ROM improvement was performed, with an empiric baseline of 89° as the average pre-treatment value across all studies and a target of 120° as the flexion required to complete activities of daily living. [17]

The attrition rate was the per-quarter subsequent-procedure rate pooled from primary review data. Health state utilities were derived from pooled EQ-5D health utility index values shared across treatment arms; the 6-moth post-treatment EQ-5D was used for the Healthy state, and the baseline EQ-5D was used for the Symptomatic/Diseased state. Utility values were shared across treatment arms due to the assumption that the quality-of-life benefit of treatment success is equivalent regardless of intervention. SAE probabilities and a conservative $3,000 per-SAE management cost were applied to all arms.

Procedural costs reflect 2026 CMS national fee schedules (Supplemental Methods S5). The ambulatory surgery center (ASC) setting was used in the main analysis. A universal hospital outpatient department (HOPD) setting and an office-based laboratory (OBL) setting for TAE, with the other two arms modeled in the ASC setting, were evaluated in sensitivity analyses (Table 1). The willingness-to-pay threshold (WTP) was $100,000/QALY. Parameter uncertainty was assessed with a 1,000-iteration probabilistic sensitivity analysis (PSA) and structural uncertainty with deterministic sensitivity analyses (DSA), as shown in Supplemental Methods S6. Analyses used R 4.4.1 with the meta, metafor, dmetar, and heemod packages.

**Table 1:** Procedure costs from a US Medicare payer perspective per treatment in each scenario defined by practice setting

Scenario	Role	TAE	MUA	ACR
All-ASC	Base case (main text)	ASC ($12,233)	ASC ($1,222)	ASC ($2,403)
TAE-OBL	Secondary (main text)	OBL ($8,135)	ASC ($1,222)	ASC ($2,403)
All-HOPD	Sensitivity (Supplement)	HOPD ($19,513)	HOPD ($1,992)	HOPD ($4,101)

## Results

### Comparative Meta-Analysis

Across 4,941 retrieved records (1,784 TAE, 1,118 MUA, and 2,039 ACR) that reduced to 2,589 unique records after duplicate removal, 10 TAE studies, 25 MUA studies, and 36 ACR studies were included (Fig. 1). Nine TAE studies were single-arm cohorts, while one was a comparative non-RCT to ACR. The MUA and ACR arms had 4 and 6 RCT arms, respectively (7 unique RCTs, of which 3 were head-to-head MUA versus ACR trials). Pain and ROM, specifically forward elevation, were the most reported outcomes across arms. Study characteristics can be seen in Tables S1-S3. Seven RCTs were assessed with RoB 2, and 55 non-randomized studies were assessed with ROBINS-I (Figure S1); per-domain traffic-light judgments are reproduced in Supplementary Figures S2–S3.

**Fig. 3:**
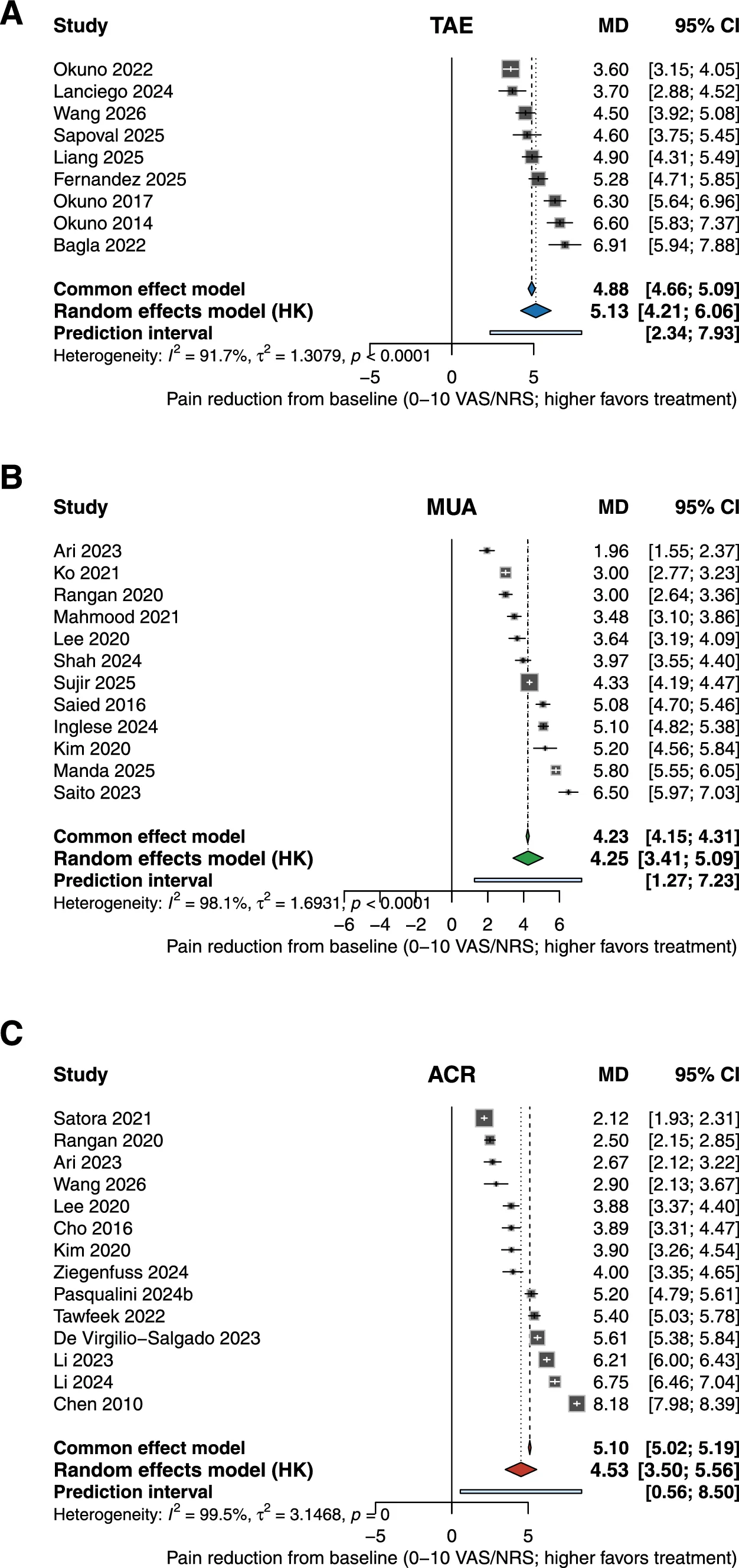
Single-arm random-effects forest plots of pain scores at 3 months. Per-arm pools combine study-level mean differences in pain VAS/NRS units (0–10). The diamond at the bottom of each panel is the random-effects pooled mean difference and the dashed horizontal band is its prediction interval. (**A**) TAE showed an average pain reduction of 5.13 points (95% CI 4.21–6.06), while (**B**) MUA showed an average pain reduction of 4.25 points (95% CI 3.41–5.09), and ACR showed an average pain reduction of 4.53 points (95% CI 3.50–5.56)

**Fig. 4:**
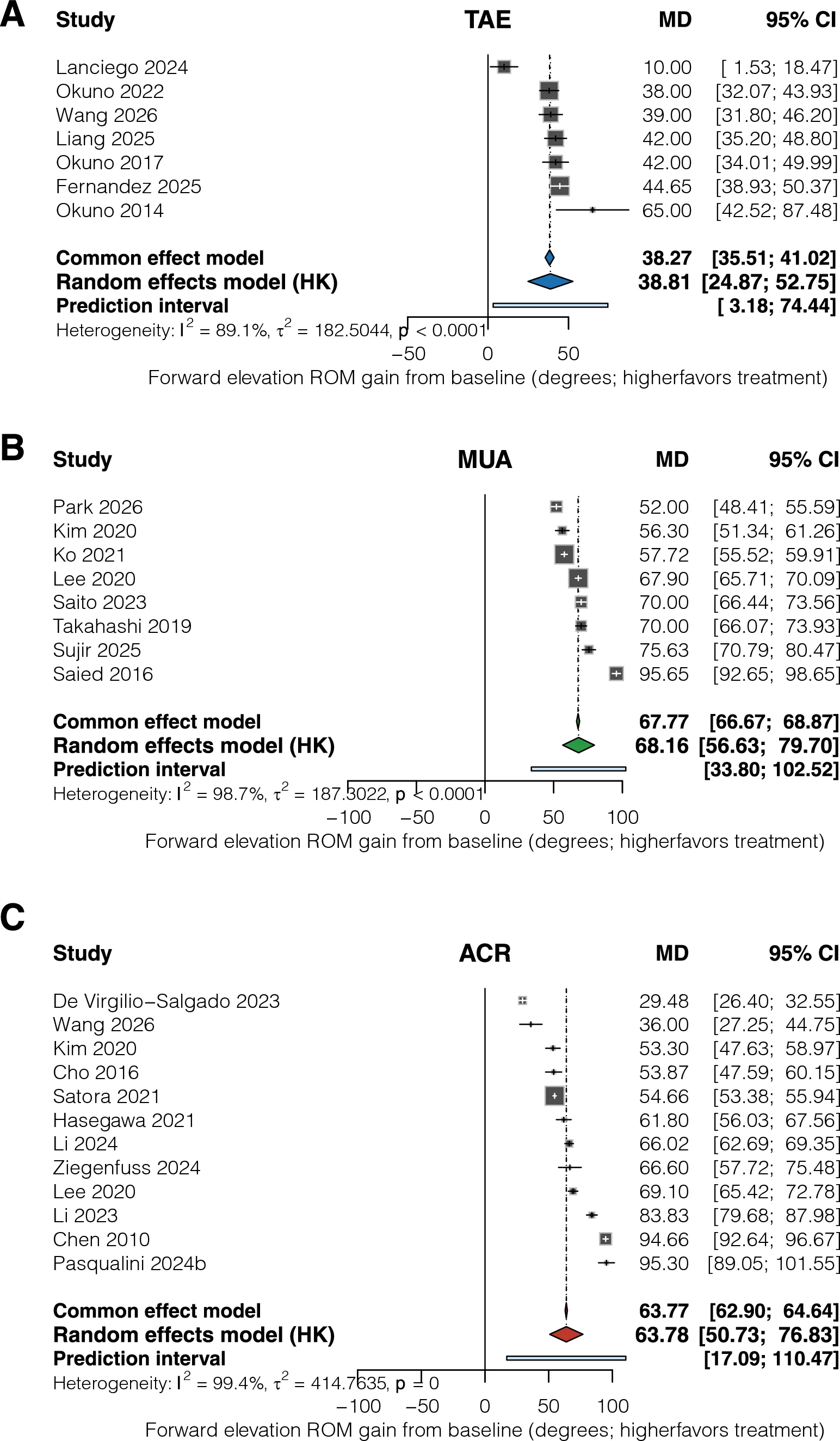
Single-arm random-effects forest plots of forward elevation ROM at 3 months. Per-arm pools combine study-level mean differences in forward elevation ROM (in degrees). The diamond at the bottom of each panel is the random-effects pooled mean difference and the dashed horizontal band is its prediction interval. (**A**) TAE showed a mean forward elevation ROM improvement of 38.81° (95% CI 24.87°-52.75°), while MUA showed an average improvement of 68.16° (95% CI 56.63°-79.70°), and ACR showed an average improvement of 63.78° (95% CI 50.73°-76.83°)

At 3 months, TAE produced the largest reductions in pain scores (MD 5.13, 95% CI 4.21–6.06), with lower reductions from ACR (4.53, 95% CI 3.50–5.56) and MUA (4.25, 95% CI 3.41–5.09, Fig. 3). TAE also showed greater improvements in the ASES functional score (45.14 vs. 42.81 for MUA and 36.98 for ACR, Figure S4). However, surgical options produced a larger increase in forward elevation ROM (MUA MD 68.16°, 95% CI 56.63°-79.70°; ACR MD 63.78°, 95% CI 50.73°-76.83°) compared to TAE (MD 38.81°, 95% CI 24.87°-52.75°, Fig. 4). Similar trends were found for external rotation ROM (Figure S5). All arms improved steadily in pain (Figure S6A) and ASES function (Figure S6D). While MUA showed the greatest early range-of-motion gains, TAE showed more gradual improvement (Figure S6B–C).

While TAE exhibited a higher rate of adverse events (pooled rate of 32.2% vs. 4.3% for MUA and 3.4% for ACR, Figure S7), no TAE study reported any SAEs, compared to several reports for MUA and ACR (0.5% and 0.4%, respectively, Figure S8). Funnel plot asymmetry suggested possible publication or small-study bias across arms and outcomes (Figures S9–S13).

### Cost-Effectiveness Analysis

Model inputs are summarized in Table 1. Procedural costs were $12,233 for TAE, $1,222 for MUA, and $2,403 for ACR in the ASC setting, with a TAE cost of $8,135 in the OBL setting and higher costs for all arms in the HOPD setting. As shown in Table 2, the pain-based clinical success probability at 3 months was highest for TAE (73.3%), followed by ACR (61.4%) and MUA (55.5%). In the secondary ROM-based analysis, MUA had the highest 3-month success probability (98.5%), followed by ACR (97.2%) and TAE (68.0%). Quarterly attrition rates from the Healthy to Diseased state, derived from pooled subsequent-procedure rates, were lowest for TAE (3 events across 4 studies, 0.5% per quarter) and highest for ACR (25 events across 4 studies, 1.8% per quarter), with MUA intermediate (18 events across 5 studies, 1.1% per quarter). Pooled health state utilities were 0.737 for the Healthy state and 0.422 for the Symptomatic and Diseased states. No SAEs were reported in the TAE literature; pooled SAE rates were 0.5% for MUA and 0.4% for ACR.

**Table 2:** Markov model input parameters, distributions, and sensitivity ranges

Parameter	Arm	Base value	Distribution	Sensitivity range (95% CI)
Clinical success (pain NRS ≤ 3 at 3 month, cycle 1)	TAE	73.3%	Beta (n = 349)	68.5%–77.8%
	MUA	55.5%	Beta (n = 894)	52.2%–58.7%
	ACR	61.4%	Beta (n = 803)	58.0%–64.7%
Clinical success (FE ≥ 120° at 3 month, cycle 1)	TAE	68.0%	Beta (n = 271)	62.3%–73.4%
	MUA	98.5%	Beta (n = 637)	97.4%–99.3%
	ACR	97.2%	Beta (n = 632)	95.8%–98.4%
Attrition, per quarter (Healthy → Diseased)	TAE	0.53%	Beta (4, 560)	0.19%–1.55%
	MUA	1.09%	Beta (19, 1629)	0.70%–1.72%
	ACR	1.81%	Beta (26, 1353)	1.24%–2.67%
Utility, Symptomatic and Diseased states (u_S, u_D)	All	0.42	Beta (n = 767)	0.39–0.46
Utility, Healthy state (u_H)	All	0.74	Beta (n = 659)	0.70–0.77
Procedure cost, ASC base (USD)	TAE	$12,233	Gamma (CV 0.25)	$6,992–$18,916
	MUA	$1,222	Gamma (CV 0.25)	$698–$1,889
	ACR	$2,403	Gamma (CV 0.25)	$1,374–$3,716
Serious adverse-event probability	TAE	0%	Beta	0.0%–1.1%
	MUA	0.5%	Beta	0.2%–1.0%
	ACR	0.4%	Beta	0.1%–0.9%
Adverse-event management cost (USD)	All	$3,000	Gamma (CV 0.25)	$1,715–$4,639
Discount rate (annual)	All	3%	Fixed	Not varied

**Fig. 5:**
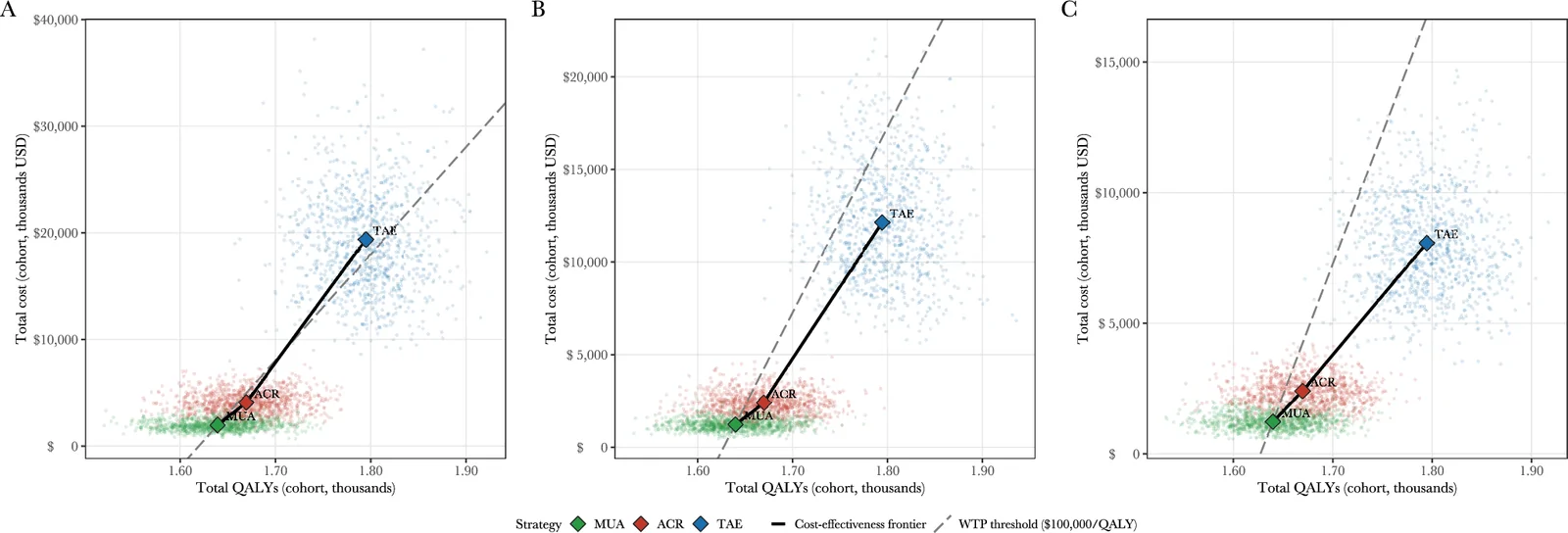
Cost-effectiveness frontier at the 36-month horizon with success defined as pain NRS ≤ 3 at 3 months. For each panel, the three diamonds are the treatment strategies plotted by total cohort cost (vertical axis, thousands USD per 1,000-patient cohort) and total QALYs (horizontal axis, thousands per cohort). The light scatter clouds are the 1,000-iteration PSA draws with sampled parameters. The black line is the cost-effectiveness frontier; the gray dashed line is the WTP of $100,000 per QALY drawn through the cheapest strategy. (**A**) In the HOPD setting, TAE exhibits a much larger total cost compared to MUA and ACR, placing it far beyond the $100,000/QALY WTP. (**B**) In the ASC setting, lower procedural costs enable TAE to sit within the WTP threshold as the most cost-effective management option. (**C**) With lowest costs in the OBL setting, TAE sits comfortably within the $100,000/QALY WTP

**Fig. 6:**
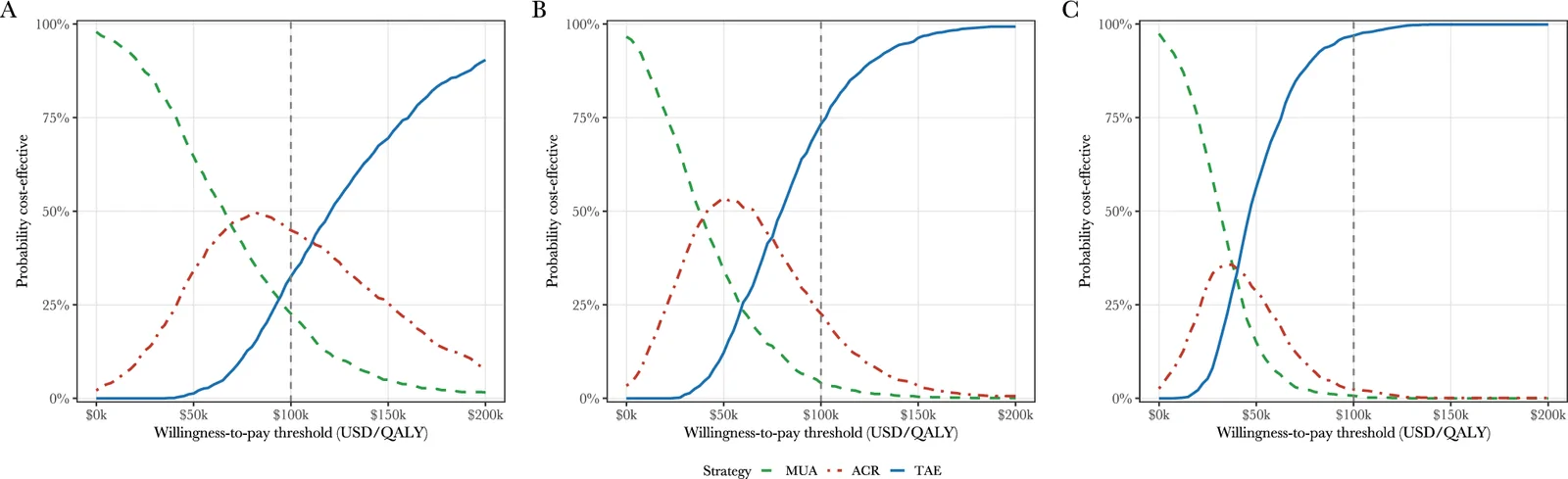
Cost-effectiveness acceptability curves at the 36-month horizon with success defined as pain NRS ≤ 3 at 3 months. Each curve indicates the cost-effectiveness probability of the treatment at a particular WTP, with the WTP of $100,000 per QALY indicated by the gray dashed line. (**A**) In the HOPD setting, ACR shows the highest cost-effectiveness probability followed by TAE and MUA. (**B**) In the ASC setting, TAE shows the highest cost-effectiveness probability followed by ACR and MUA. (**C**) In the OBL setting, TAE shows the highest cost-effectiveness probability with greater separation from ACR and MUA

Over a 36-month horizon per 1,000 patients, TAE produced the most QALYs (1,795) at the highest total cost ($12,143,173), while MUA produced the fewest QALYs (1,640) at the lowest cost ($1,227,456); ACR was intermediate on both dimensions (1,669 QALYs; $2,397,517, Table 3). All three strategies fell on the cost-effectiveness frontier (Fig. 5). The incremental cost-effectiveness ratio (ICER) of TAE versus ACR was $77,807/QALY (Table 3). At a WTP threshold of $100,000/QALY, TAE was the optimal strategy, producing the highest net monetary benefit ($167,313,368 per 1,000 patients) with a cost-effectiveness probability of approximately 73%, followed by ACR ($164,533,619, 23%) and MUA ($162,733,046, 4%), as shown in Table 3 and Fig. 6B. When TAE was modeled in the OBL setting at a lower procedural cost of $8,135, the ICER of TAE versus ACR decreased further to $45,327/QALY, and the probability of TAE being cost-effective at $100,000/QALY increased to approximately 97% (Figs. 5C and 6C). Modeling the three arms in a HOPD setting increased costs and resulted in TAE no longer being the optimal strategy at the $100,000/QALY WTP, with an ICER of $122,039 versus ACR and cost-effectiveness probability of 33% (Figs. 5A and 6A). Multiway DSA shows substantial sensitivity to TAE’s clinical success rate, with minimal sensitivity to TAE’s attrition rate (Figure S14). A leave-one-out sensitivity analysis sequentially excluding each TAE study confirmed that TAE remains the optimal strategy for 8 of 9 iterations in the ASC setting and all iterations in the OBL setting (Figure S15). [18]

**Table 3:** Cost-effectiveness results for success defined by pain reduction across all three practice settings Within each setting, strategies are sorted by total cost; ICERs are computed sequentially along the cost-effectiveness frontier (each non-dominated strategy vs the next-cheaper non-dominated strategy, with incremental cost and QALYs reported against that same comparator). NMB, net monetary benefit at the $100,000/QALY threshold; CE probability, % of 1,000 PSA draws with the highest NMB

Setting	Strategy	Total cost (USD)	QALYs	Incremental cost (USD)	Incremental QALYs	ICER (USD/QALY)	NMB (USD)	CE probability
HOPD	MUA	1,991,737	1,639.6	0	0.00	— (reference)	161,968,764	22.6%
	ACR	4,083,016	1,669.3	2,091,279	29.71	70,398	162,848,121	44.9%
	TAE	19,368,949	1,794.6	15,285,933	125.25	122,039	160,087,592	32.5%
ASC	MUA	1,227,456	1,639.6	0	0.00	— (reference)	162,733,046	4.1%
	ACR	2,397,517	1,669.3	1,170,062	29.71	39,388	164,533,619	22.6%
	TAE	12,143,173	1,794.6	9,745,656	125.25	77,807	167,313,368	73.3%
OBL	MUA	1,227,456	1,639.6	0	0.00	— (reference)	162,733,046	0.7%
	ACR	2,397,517	1,669.3	1,170,062	29.71	39,388	164,533,619	2.4%
	TAE	8,074,898	1,794.6	5,677,380	125.25	45,327	171,381,644	96.9%

**Fig. 7:**
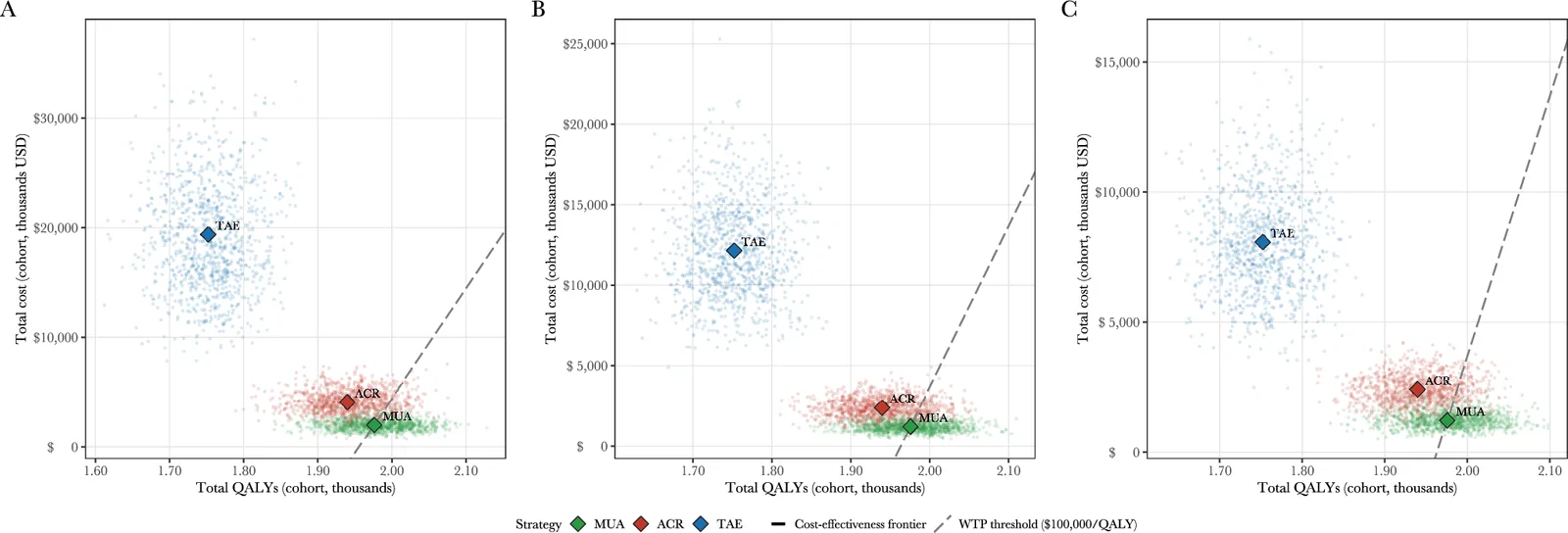
Cost-effectiveness frontier at the 36-month horizon with success defined as forward elevation ROM ≥ 120° at 3 months. For each panel, the three diamonds are the treatment strategies plotted by total cohort cost (vertical axis, thousands USD per 1,000-patient cohort) and total QALYs (horizontal axis, thousands per cohort). The light scatter clouds are the 1,000-iteration PSA draws with sampled parameters. MUA achieved greater ROM improvement at lower cost than ACR and TAE, so the cost-effectiveness frontier collapses to MUA alone and no frontier line is drawn; the gray dashed line is the WTP of $100,000 per QALY. This holds across (**A**) HOPD, (**B**) ASC, and (**C**) OBL settings

When clinical success was instead defined by forward elevation ROM ≥ 120° at 3 months, TAE produced fewer QALYs and higher cost in all practice settings (Fig. 7), reflecting the smaller ROM improvement observed with TAE (MD 38.8°) compared with MUA (MD 68.2°) and ACR (MD 63.8°, Fig. 3). MUA produced 1,975 QALYs per 1,000 patients at a cost of $1,227,456, compared with 1,939 QALYs at $2,397,517 for ACR and 1,752 QALYs at $12,143,173 for TAE (Table S4).

## Discussion

This study represents the first CEA comparing TAE with MUA and ACR for primary AC refractory to conservative management. The principal finding is that TAE is cost-effective compared to both surgical alternatives when the clinical objective is pain relief, driven by a higher probability of achieving mild pain at 3 months and lower rates of SAEs and subsequent procedures. Modeling TAE in the lower-cost OBL setting further increased its cost-effectiveness compared to the ASC and HOPD settings. However, when clinical success was defined by ROM recovery, MUA and ACR yielded more QALYs at lower cost, thus dominating the cost-effectiveness frontier over TAE.

The outcome-dependent divergence aligns with AC’s underlying mechanism. Contemporary models describe an inflammatory arm characterized by synovial angiogenesis and neoinnervation that drives nociceptive pain in addition to a fibrotic arm characterized by capsular contracture that drives mechanical restriction [19,20]. TAE, by devascularizing periarticular neovascularity and their accompanying sensory nerves, addresses the nociceptive driver but does not directly address capsular fibrosis [21,22]. Shintaku et al. demonstrated on FDG-PET/CT that chronic periarticular inflammation decreased significantly after TAE and that the magnitude of FDG uptake reduction correlated with pain improvement [22]. Conversely, MUA and ACR mechanically disrupt mechanical contracture, accounting for their larger ROM gains. [23,24 ]

This divergence raises a clinically important question: does pain or ROM matter more in AC? Patients present along a spectrum from pain-predominant to stiffness-predominant disease; classically, early restriction is substantially pain-mediated, whereas later restriction reflects fixed contracture [20, 25-27]. In qualitative studies, patients ultimately identify functional recovery and pain relief as intertwined treatment priorities [28–31]. Additionally, pain reduction was selected as a secondary outcome for the UK FroST, and a core outcome set identified functional status and pain levels as the most cited primary outcomes by healthcare professionals [15,32 ]. Taken together, these data suggest that pain and ROM are not competing priorities, but rather complementary concerns that may temporally evolve along the disease trajectory.

This temporal evolution of patient priorities aligns with evidence that treatment efficacy in AC is moderated by disease stage. Zhang et al., in a network meta-analysis of 92 trials (5,946 patients), found that steroid injection provided more benefit during the freezing phase, whereas mechanical interventions provided more benefit in the adhesive phase, a phase-dependent pattern that may extend to procedural comparators [33]. In the early freezing phase, when inflammation and pain dominate, TAE may offer the best value by targeting abnormal periarticular neovascularity without requiring general anesthesia [21,22 ]. In the later frozen phase, when fibrotic contracture predominates, MUA or ACR may be more appropriate given their superior ROM gains. TAE may also be well-suited for patients who are poor candidates for general anesthesia or for patients who are especially risk-averse, given that no SAEs were reported across studies.

The finding that MUA is the most cost-effective intervention for ROM improvement aligns with prior analyses. The UK FroST found MUA to be the most cost-effective option within the NHS at a £20,000/QALY threshold, and Sundararajan et al. reported that MUA was more cost-effective than ACR with equivalent clinical outcomes [15,34 ]. The present analysis extends this literature by incorporating TAE as a third comparator from a U.S. Medicare payer perspective, finding that TAE’s higher procedural cost is offset by superior pain relief. Collectively, these findings highlight a tradeoff between pain-focused and ROM-focused value propositions, suggesting that treatment selection for adhesive capsulitis should be guided by patient’s preferences along the pain-to-stiffness continuum.

Several limitations warrant consideration. The TAE evidence base consists almost entirely of single-arm cohort studies, with no randomized comparisons to MUA or ACR, requiring success criteria to be derived from comparative single-arm meta-analyses rather than a network meta-analysis. No studies were excluded on risk-of-bias ratings given the limited evidence pool; instead, a leave-one-out sensitivity analysis was performed to bound the influence of any single TAE study. The included studies likely enrolled patients at different stages of disease progression, and because individual patient data on disease stage were unavailable, part of the observed between-arm differences may reflect patient selection rather than true treatment efficacy. Future prospective studies stratifying by symptom duration would be needed to validate the stage-specific treatment selection framework proposed herein.

The Markov model carries several limitations. For instance, it assumes homogeneous transition probabilities within each arm without stratification by disease phase. It also assumes no spontaneous recovery and captures attrition only through subsequent-procedure rates, a conservative structure consistent with the refractory cohort but one that may disadvantage arms with lower early success. Whether patients who underwent re-intervention were truly initial responders cannot be ascertained, and unmeasured sources of treatment failure are not modeled. Re-intervention costs were excluded because the type of subsequent procedure was heterogeneous and often unspecified, precluding reliable cost assignment. Few studies reported long-term outcomes, necessitating extrapolation over the 36-month horizon. A 12-month sensitivity analysis was performed (Figure S15), though this shorter time horizon constrains the interval over which outcome separations can accumulate. Costs reflect U.S. Medicare fee schedules and may not generalize to other payer systems. Finally, the anchored threshold formulation, while methodologically justified for handling baseline heterogeneity, introduces dependence on the UK FroST reference baseline, and alternative success definitions may influence the absolute ICER.^12,15^ Comparative RCTs and expansion of the TAE evidence base would allow more robust model refinement.

## Conclusions

TAE was cost-effective compared with MUA and ACR for refractory adhesive capsulitis at a $100,000/QALY threshold when clinical success was defined by pain relief. When success was defined by ROM recovery, surgical approaches yielded more QALYs at lower cost. Based on indirect comparisons, these findings may support the value of TAE as a minimally invasive, cost-effective alternative for pain-predominant refractory adhesive capsulitis.

## Supplementary Information

Below is the link to the electronic supplementary material.

Supplementary file1 (PNG 573 kb)

Supplementary file2 (PNG 203 kb)

Supplementary file3 (PNG 1321 kb)

Supplementary file4 (PDF 7 kb)

Supplementary file5 (PDF 8 kb)

Supplementary file6 (PDF 400 kb)

Supplementary file7 (PDF 10 kb)

Supplementary file8 (PDF 9 kb)

Supplementary file9 (PDF 168 kb)

Supplementary file10 (PDF 166 kb)

Supplementary file11 (PDF 154 kb)

Supplementary file12 (PDF 149 kb)

Supplementary file13 (PDF 148 kb)

Supplementary file14 (PDF 271 kb)

Supplementary file15 (PDF 280 kb)

Supplementary file16 (PDF 886 kb)

Supplementary file17 (DOCX 14 kb)

Supplementary file18 (DOCX 15 kb)

Supplementary file19 (DOCX 16 kb)

Supplementary file20 (DOCX 17 kb)

Supplementary file21 (DOCX 62 kb)
