# Supplementary material for: Transarterial Embolization Versus Manipulation Under Anesthesia and Arthroscopic Capsular Release for Refractory Adhesive Capsulitis—A Cost-Effectiveness Analysis
Source: Cardiovasc Intervent Radiol. 2026 Aug 2;49(10):1977–88. doi: 10.1007/s00270-026-04563-8 (PMC13642511; doi:10.1007/s00270-026-04563-8)
Supplement: Supplementary file 2 [file 270_2026_4563_MOESM11_ESM.pdf]

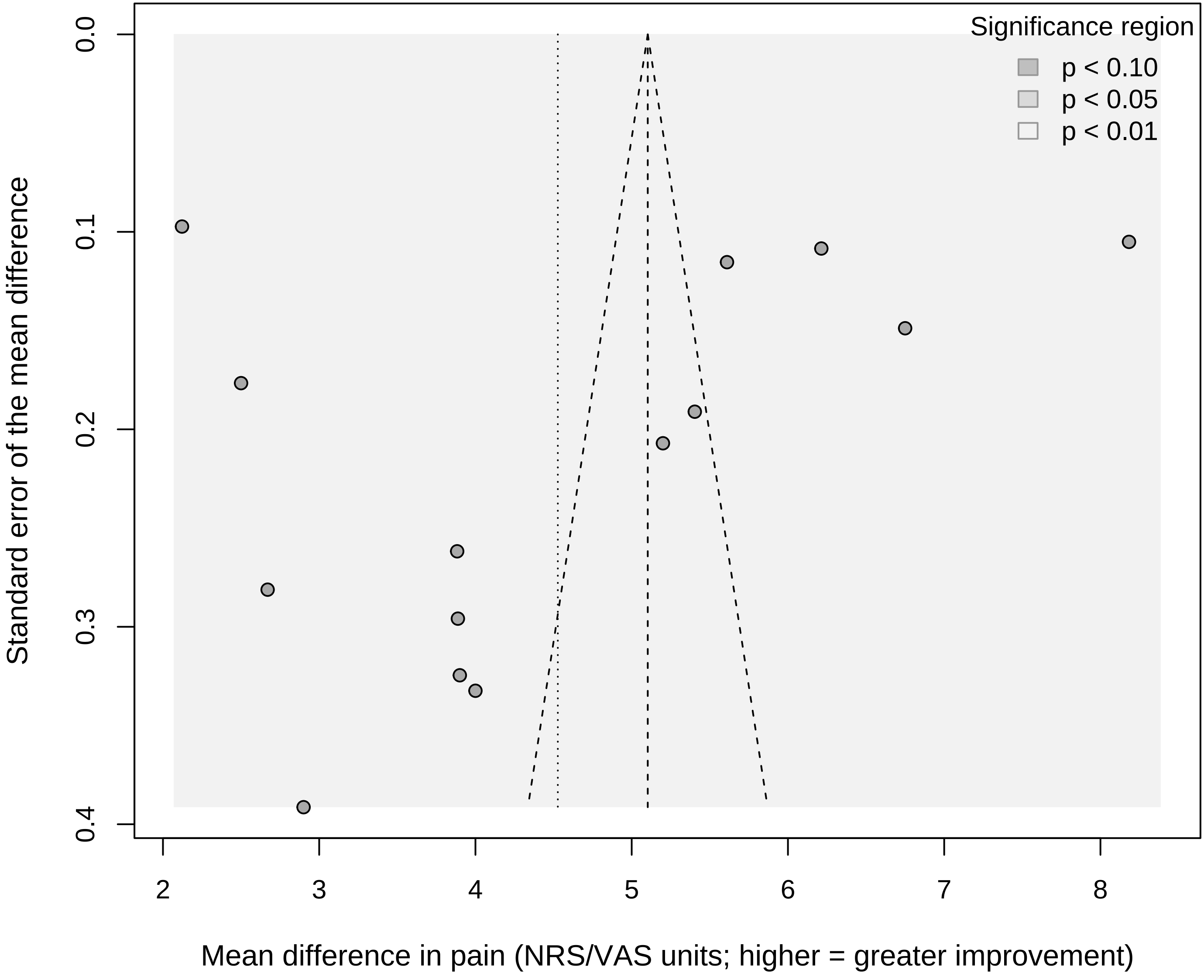
Significance region
p < 0.10
p < 0.05
p < 0.01
Standard error of the mean difference
0.0
0.1
0.2
0.3
0.4
2
3
4
5
6
7
8
Mean difference in pain (NRS/VAS units; higher = greater improvement)
