# Supplementary material for: Transarterial Embolization Versus Manipulation Under Anesthesia and Arthroscopic Capsular Release for Refractory Adhesive Capsulitis—A Cost-Effectiveness Analysis
Source: Cardiovasc Intervent Radiol. 2026 Aug 2;49(10):1977–88. doi: 10.1007/s00270-026-04563-8 (PMC13642511; doi:10.1007/s00270-026-04563-8)
Supplement: Supplementary file 3 [file 270_2026_4563_MOESM12_ESM.pdf]

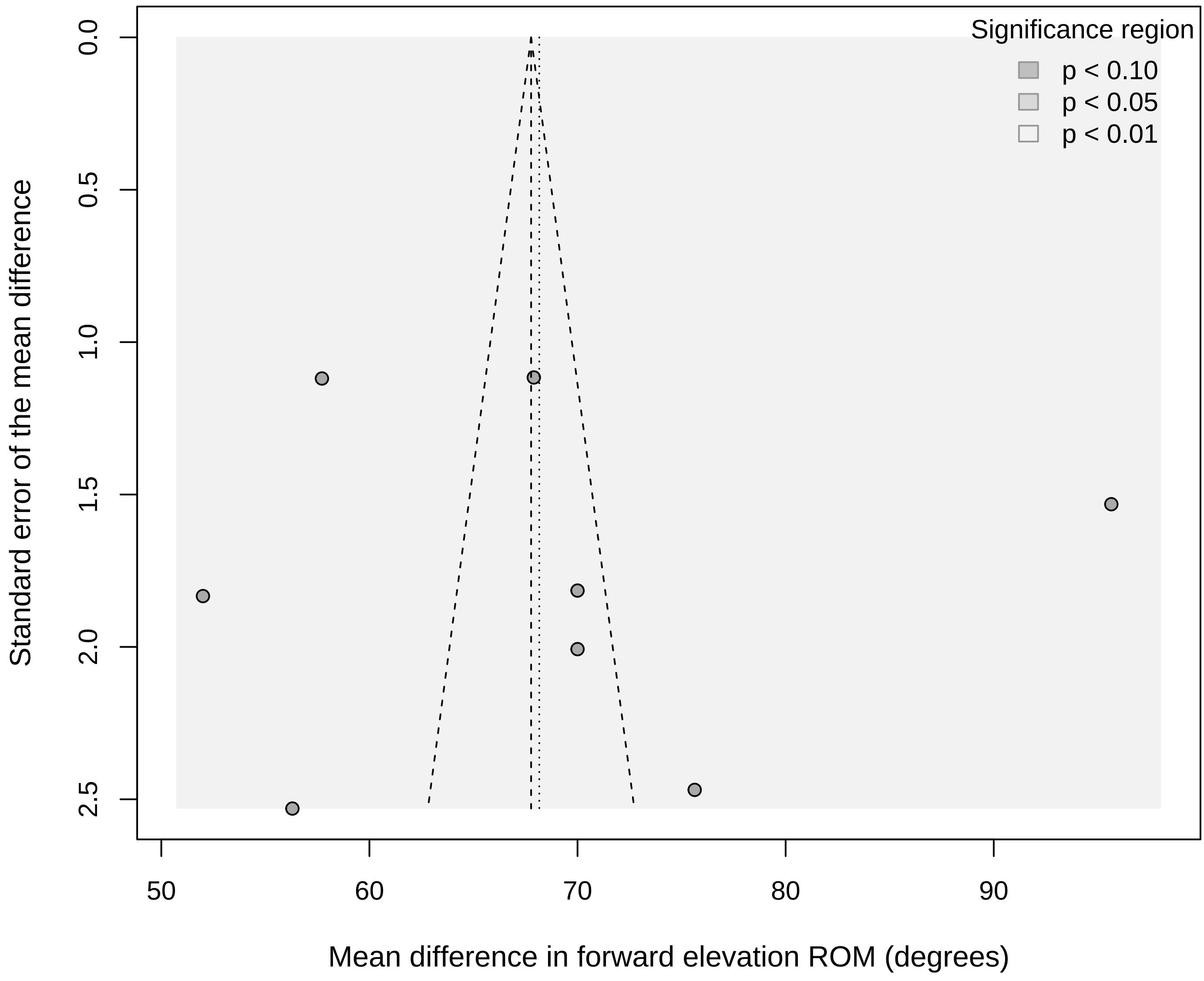
Significance region
p < 0.10
p < 0.05
p < 0.01
Standard error of the mean difference
0.0
0.5
1.0
1.5
2.0
2.5
50
60
70
80
90
Mean difference in forward elevation ROM (degrees)
