# Supplementary material for: Transarterial Embolization Versus Manipulation Under Anesthesia and Arthroscopic Capsular Release for Refractory Adhesive Capsulitis—A Cost-Effectiveness Analysis
Source: Cardiovasc Intervent Radiol. 2026 Aug 2;49(10):1977–88. doi: 10.1007/s00270-026-04563-8 (PMC13642511; doi:10.1007/s00270-026-04563-8)
Supplement: Supplementary file 5 [file 270_2026_4563_MOESM14_ESM.pdf]

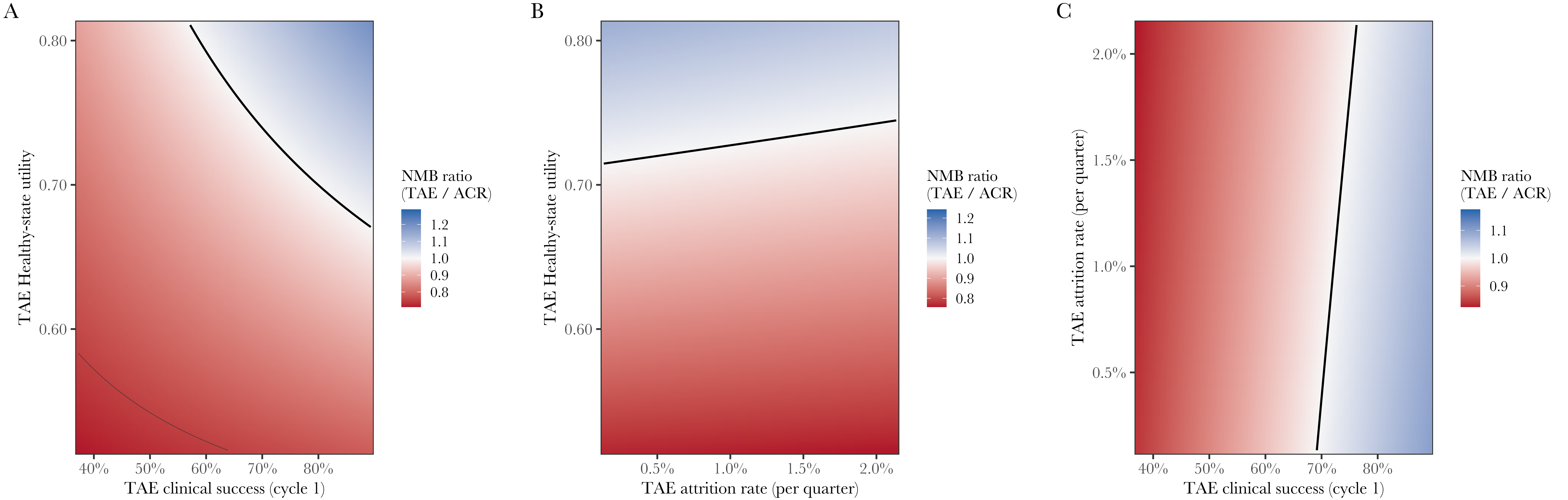
A
TAE Healthy-state utility
0.80
0.70
0.60
40%
50%
60%
70%
80%
TAE clinical success (cycle 1)
NMB ratio
(TAE / ACR)
1.2
1.1
1.0
0.9
0.8
B
TAE Healthy-state utility
0.80
0.70
0.60
0.5%
1.0%
1.5%
2.0%
TAE attrition rate (per quarter)
NMB ratio
(TAE / ACR)
1.2
1.1
1.0
0.9
0.8
C
TAE attrition rate (per quarter)
2.0%
1.5%
1.0%
0.5%
40%
50%
60%
70%
80%
TAE clinical success (cycle 1)
NMB ratio
(TAE / ACR)
1.1
1.0
0.9
