# Supplementary material for: Transarterial Embolization Versus Manipulation Under Anesthesia and Arthroscopic Capsular Release for Refractory Adhesive Capsulitis—A Cost-Effectiveness Analysis
Source: Cardiovasc Intervent Radiol. 2026 Aug 2;49(10):1977–88. doi: 10.1007/s00270-026-04563-8 (PMC13642511; doi:10.1007/s00270-026-04563-8)
Supplement: Supplementary file 6 [file 270_2026_4563_MOESM15_ESM.pdf]

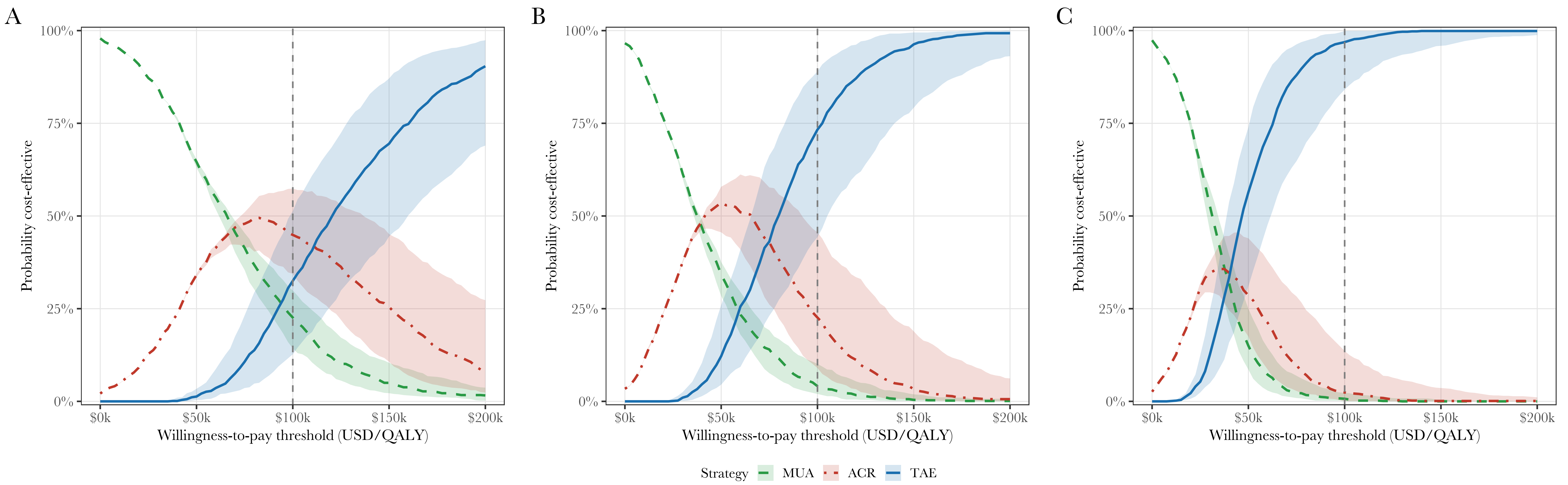
A
B
C
Probability cost-effective
100%
75%
50%
25%
0%
$0k
$50k
$100k
$150k
$200k
Willingness-to-pay threshold (USD/QALY)
Strategy
MUA
ACR
TAE
