# Supplementary material for: Transarterial Embolization Versus Manipulation Under Anesthesia and Arthroscopic Capsular Release for Refractory Adhesive Capsulitis—A Cost-Effectiveness Analysis
Source: Cardiovasc Intervent Radiol. 2026 Aug 2;49(10):1977–88. doi: 10.1007/s00270-026-04563-8 (PMC13642511; doi:10.1007/s00270-026-04563-8)
Supplement: Supplementary file 7 [file 270_2026_4563_MOESM16_ESM.pdf]

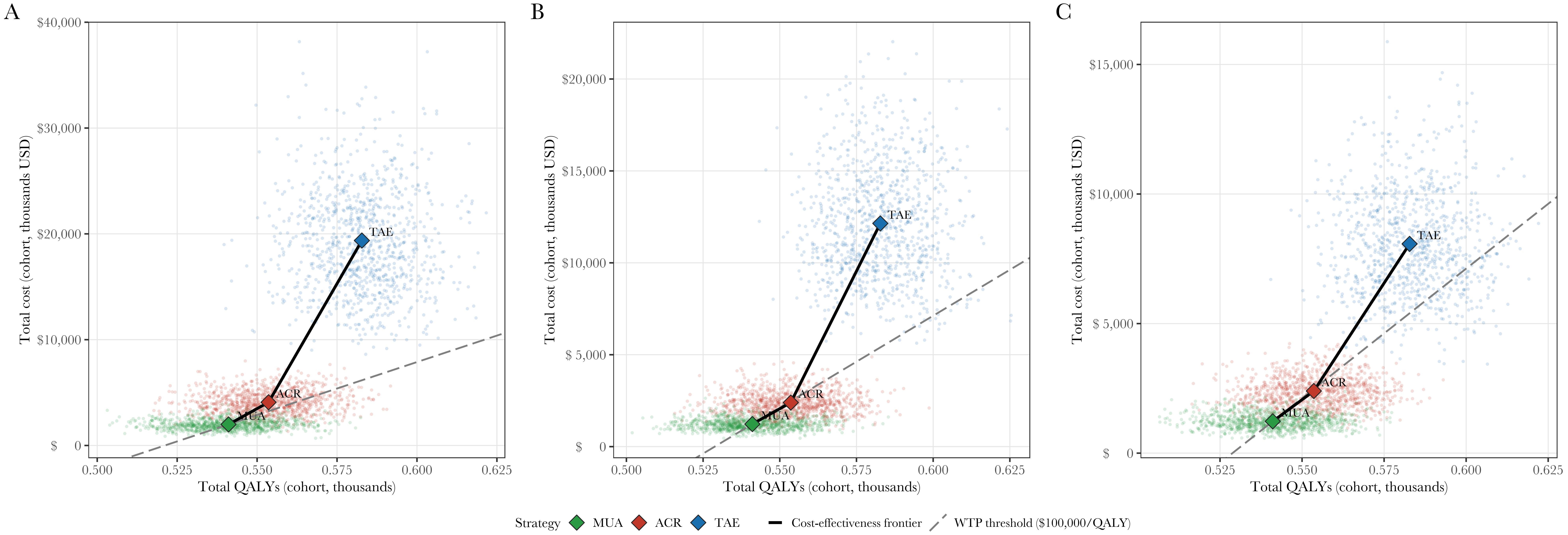
A
B
C
Total cost (cohort, thousands USD)
Total QALYs (cohort, thousands)
$40,000
$30,000
$20,000
$10,000
$ 0
$20,000
$15,000
$10,000
$ 5,000
$15,000
$10,000
$ 5,000
0.500
0.525
0.550
0.575
0.600
0.625
MUA
ACR
TAE
Strategy
Cost-effectiveness frontier
WTP threshold ($100,000/QALY)
