# Supplementary material for: Transarterial Embolization Versus Manipulation Under Anesthesia and Arthroscopic Capsular Release for Refractory Adhesive Capsulitis—A Cost-Effectiveness Analysis
Source: Cardiovasc Intervent Radiol. 2026 Aug 2;49(10):1977–88. doi: 10.1007/s00270-026-04563-8 (PMC13642511; doi:10.1007/s00270-026-04563-8)
Supplement: Supplementary file 8 [file 270_2026_4563_MOESM4_ESM.pdf]

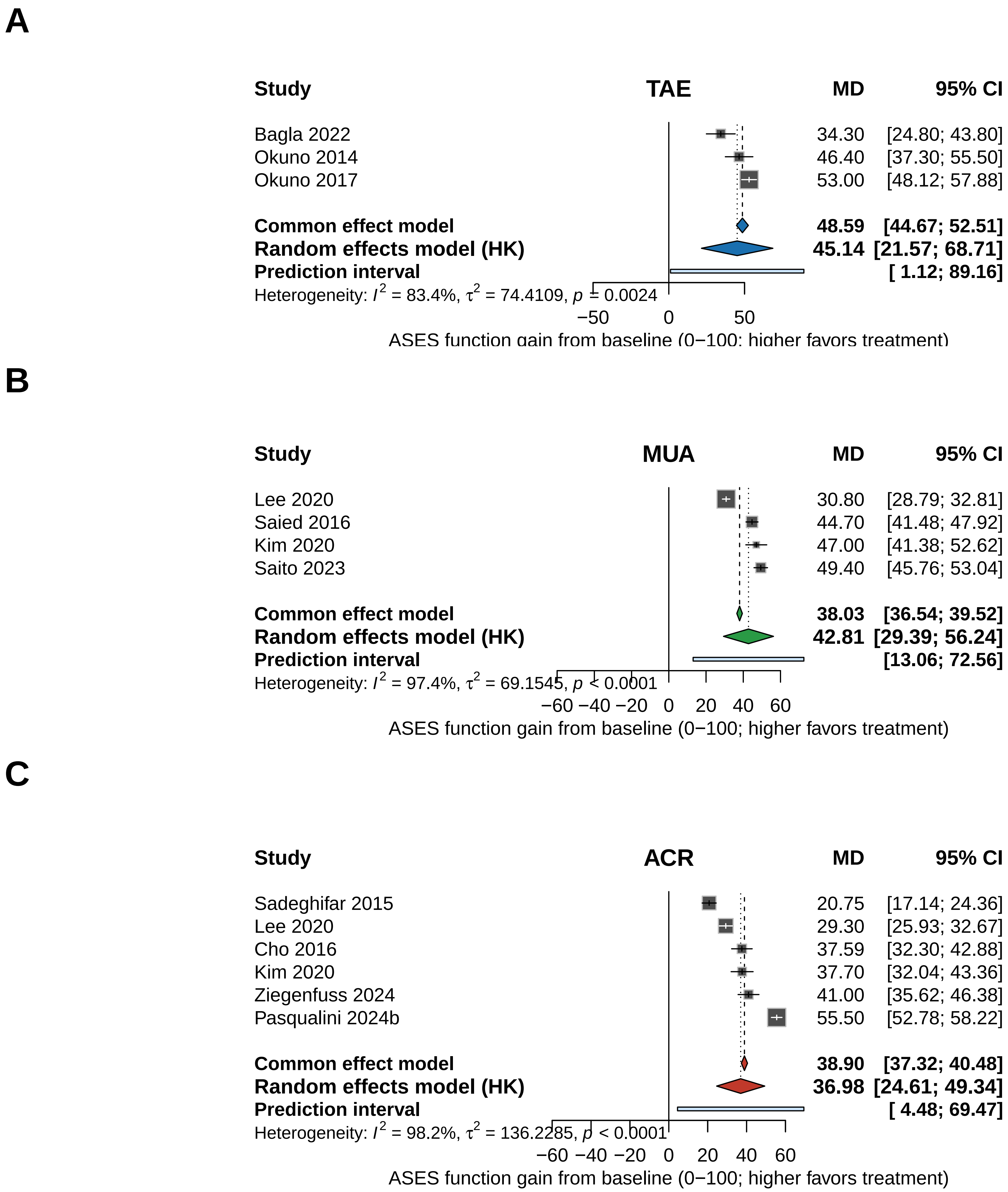
A
Study
TAE
MD
95% CI
Bagla 2022
34.30
[24.80; 43.80]
Okuno 2014
46.40
[37.30; 55.50]
Okuno 2017
53.00
[48.12; 57.88]
Common effect model
48.59
[44.67; 52.51]
Random effects model (HK)
45.14
[21.57; 68.71]
Prediction interval
[1.12; 89.16]
Heterogeneity: I^2 = 83.4%, τ^2 = 74.4109, p = 0.0024
−50
0
50
ASES function gain from baseline (0−100; higher favors treatment)
B
Study
MUA
MD
95% CI
Lee 2020
30.80
[28.79; 32.81]
Saied 2016
44.70
[41.48; 47.92]
Kim 2020
47.00
[41.38; 52.62]
Saito 2023
49.40
[45.76; 53.04]
Common effect model
38.03
[36.54; 39.52]
Random effects model (HK)
42.81
[29.39; 56.24]
Prediction interval
[13.06; 72.56]
Heterogeneity: I^2 = 97.4%, τ^2 = 69.1545, p < 0.0001
−60 −40 −20 0 20 40 60
ASES function gain from baseline (0−100; higher favors treatment)
C
Study
ACR
MD
95% CI
Sadeghifar 2015
20.75
[17.14; 24.36]
Lee 2020
29.30
[25.93; 32.67]
Cho 2016
37.59
[32.30; 42.88]
Kim 2020
37.70
[32.04; 43.36]
Ziegenfuss 2024
41.00
[35.62; 46.38]
Pasqualini 2024b
55.50
[52.78; 58.22]
Common effect model
38.90
[37.32; 40.48]
Random effects model (HK)
36.98
[24.61; 49.34]
Prediction interval
[4.48; 69.47]
Heterogeneity: I^2 = 98.2%, τ^2 = 136.2285, p < 0.0001
−60 −40 −20 0 20 40 60
ASES function gain from baseline (0−100; higher favors treatment)
