# Supplementary material for: Transarterial Embolization Versus Manipulation Under Anesthesia and Arthroscopic Capsular Release for Refractory Adhesive Capsulitis—A Cost-Effectiveness Analysis
Source: Cardiovasc Intervent Radiol. 2026 Aug 2;49(10):1977–88. doi: 10.1007/s00270-026-04563-8 (PMC13642511; doi:10.1007/s00270-026-04563-8)
Supplement: Supplementary file 9 [file 270_2026_4563_MOESM5_ESM.pdf]

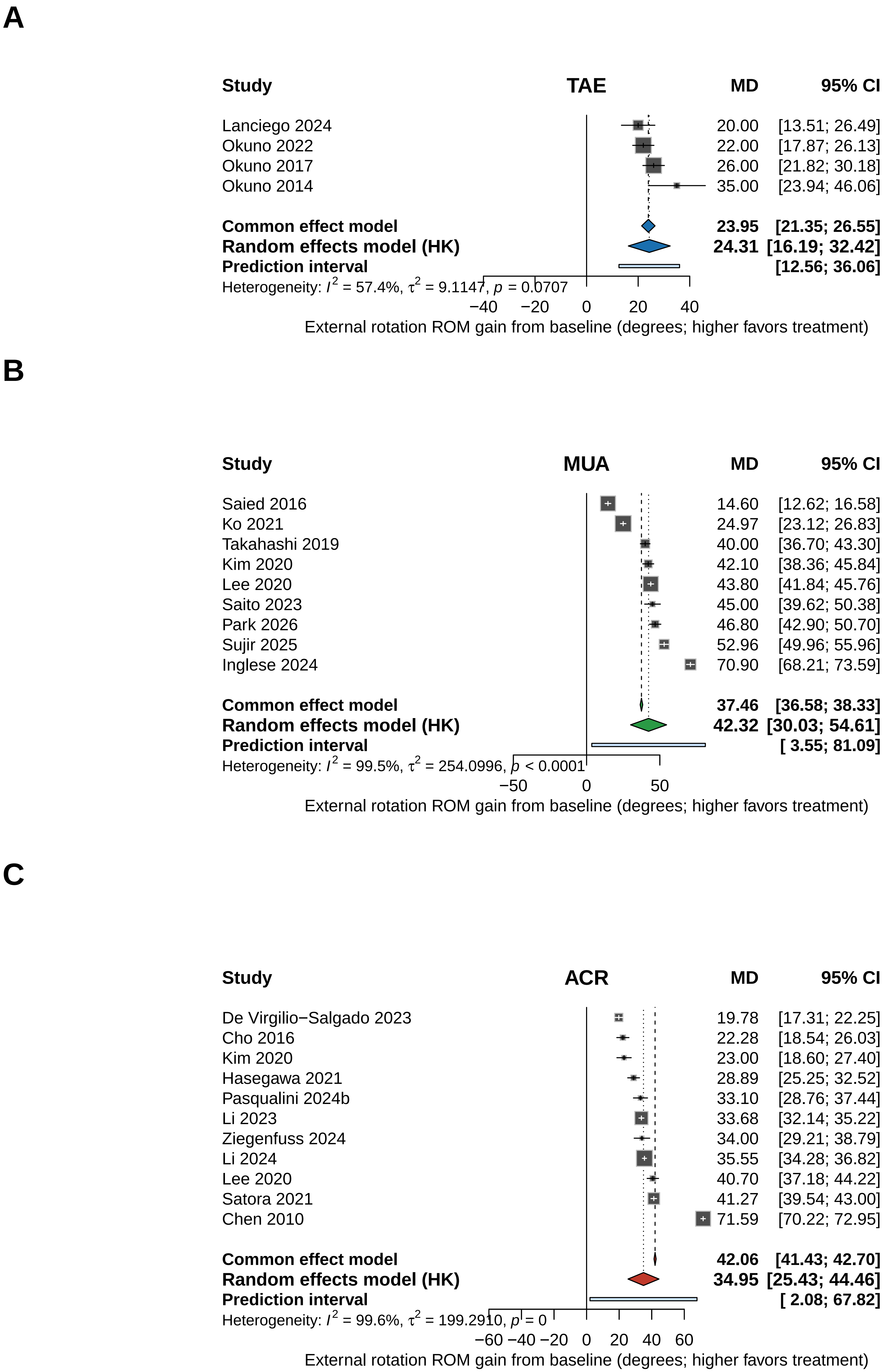
A
Study
TAE
MD
95% CI
Lanciego 2024 20.00 [13.51; 26.49]
Okuno 2022 22.00 [17.87; 26.13]
Okuno 2017 26.00 [21.82; 30.18]
Okuno 2014 35.00 [23.94; 46.06]
Common effect model 23.95 [21.35; 26.55]
Random effects model (HK) 24.31 [16.19; 32.42]
Prediction interval [12.56; 36.06]
Heterogeneity: I^2 = 57.4%, τ^2 = 9.1147, p = 0.0707
−40 −20 0 20 40
External rotation ROM gain from baseline (degrees; higher favors treatment)
B
Study
MUA
MD
95% CI
Saied 2016 14.60 [12.62; 16.58]
Ko 2021 24.97 [23.12; 26.83]
Takahashi 2019 40.00 [36.70; 43.30]
Kim 2020 42.10 [38.36; 45.84]
Lee 2020 43.80 [41.84; 45.76]
Saito 2023 45.00 [39.62; 50.38]
Park 2026 46.80 [42.90; 50.70]
Sujir 2025 52.96 [49.96; 55.96]
Inglese 2024 70.90 [68.21; 73.59]
Common effect model 37.46 [36.58; 38.33]
Random effects model (HK) 42.32 [30.03; 54.61]
Prediction interval [3.55; 81.09]
Heterogeneity: I^2 = 99.5%, τ^2 = 254.0996, p < 0.0001
−50 0 50
External rotation ROM gain from baseline (degrees; higher favors treatment)
C
Study
ACR
MD
95% CI
De Virgilio−Salgado 2023 19.78 [17.31; 22.25]
Cho 2016 22.28 [18.54; 26.03]
Kim 2020 23.00 [18.60; 27.40]
Hasegawa 2021 28.89 [25.25; 32.52]
Pasqualini 2024b 33.10 [28.76; 37.44]
Li 2023 33.68 [32.14; 35.22]
Ziegenfuss 2024 34.00 [29.21; 38.79]
Li 2024 35.55 [34.28; 36.82]
Lee 2020 40.70 [37.18; 44.22]
Satora 2021 41.27 [39.54; 43.00]
Chen 2010 71.59 [70.22; 72.95]
Common effect model 42.06 [41.43; 42.70]
Random effects model (HK) 34.95 [25.43; 44.46]
Prediction interval [2.08; 67.82]
Heterogeneity: I^2 = 99.6%, τ^2 = 199.2910, p = 0
−60 −40 −20 0 20 40 60
External rotation ROM gain from baseline (degrees; higher favors treatment)
