# Supplementary material for: Transarterial Embolization Versus Manipulation Under Anesthesia and Arthroscopic Capsular Release for Refractory Adhesive Capsulitis—A Cost-Effectiveness Analysis
Source: Cardiovasc Intervent Radiol. 2026 Aug 2;49(10):1977–88. doi: 10.1007/s00270-026-04563-8 (PMC13642511; doi:10.1007/s00270-026-04563-8)
Supplement: Supplementary file 10 [file 270_2026_4563_MOESM6_ESM.pdf]

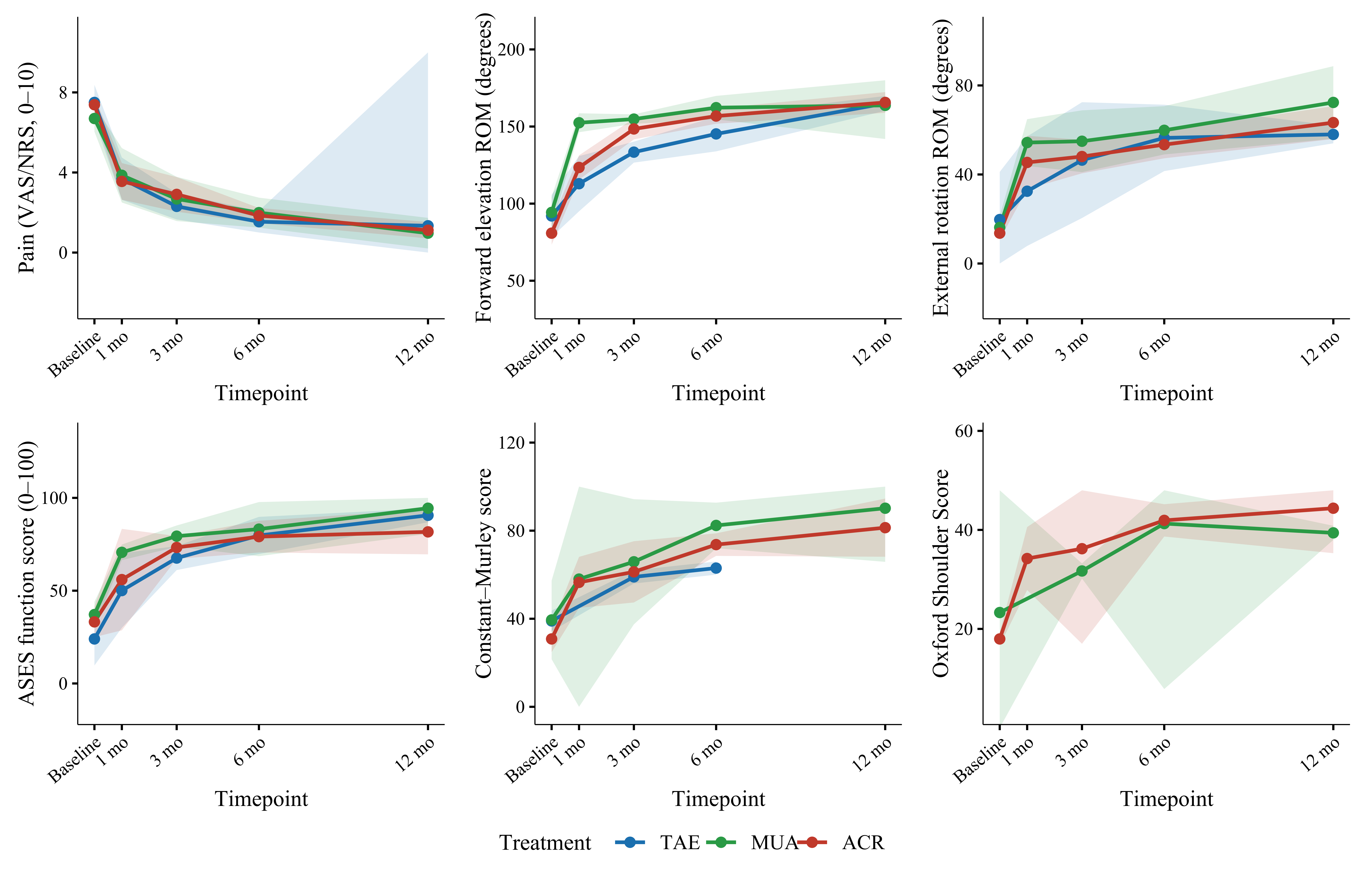

Pain (VAS/NRS, 0–10)
Forward elevation ROM (degrees)
External rotation ROM (degrees)
ASES function score (0–100)
Constant–Murley score
Oxford Shoulder Score
Baseline
1 mo
3 mo
6 mo
12 mo
Timepoint
Treatment
TAE
MUA
ACR
