# Supplementary material for: Transarterial Embolization Versus Manipulation Under Anesthesia and Arthroscopic Capsular Release for Refractory Adhesive Capsulitis—A Cost-Effectiveness Analysis
Source: Cardiovasc Intervent Radiol. 2026 Aug 2;49(10):1977–88. doi: 10.1007/s00270-026-04563-8 (PMC13642511; doi:10.1007/s00270-026-04563-8)
Supplement: Supplementary file 11 [file 270_2026_4563_MOESM7_ESM.pdf]

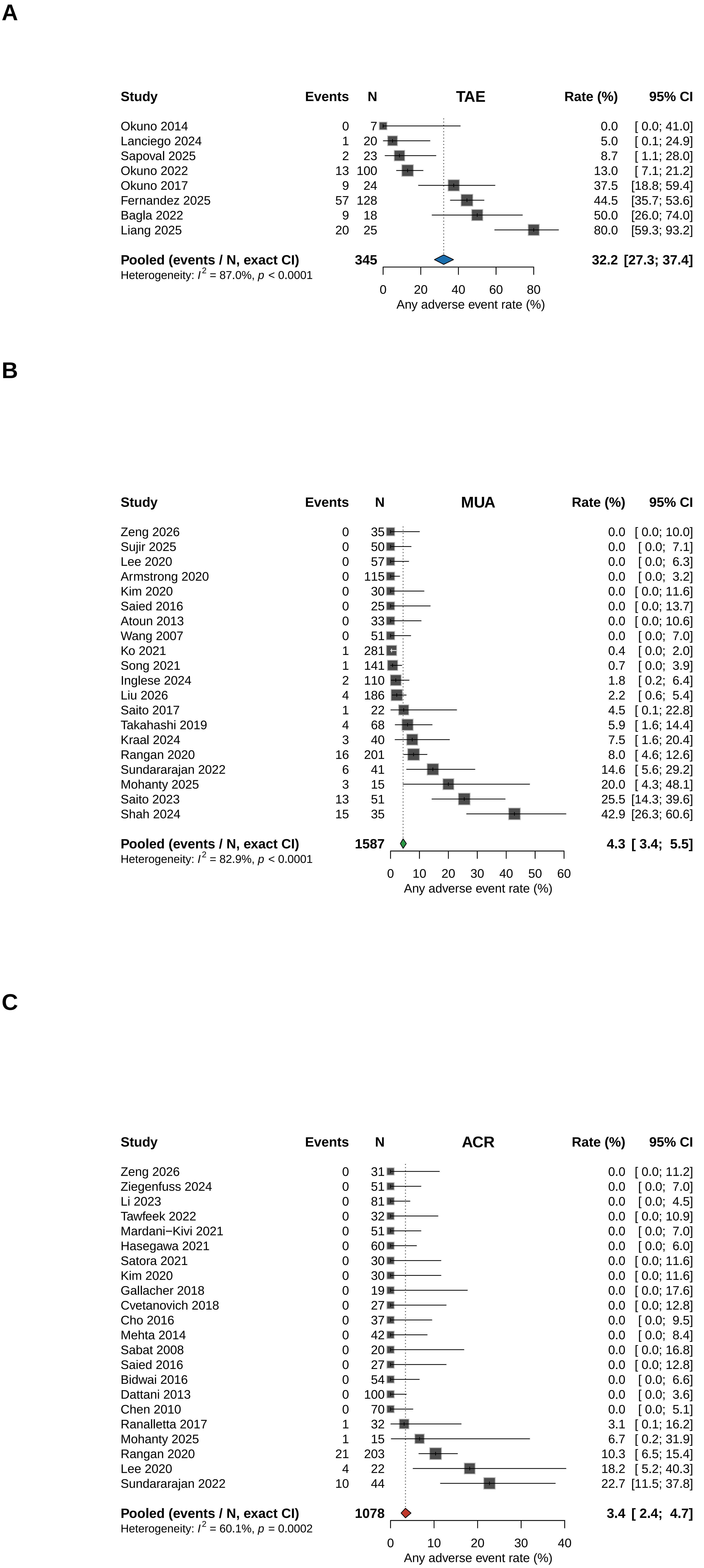
A
Study Events N TAE Rate (%) 95% CI
Okuno 2014 0 7 0.0 [0.0; 41.0]
Lanciego 2024 1 20 5.0 [0.1; 24.9]
Sapoval 2025 2 23 8.7 [1.1; 28.0]
Okuno 2022 13 100 13.0 [7.1; 21.2]
Okuno 2017 9 24 37.5 [18.8; 59.4]
Fernandez 2025 57 128 44.5 [35.7; 53.6]
Bagla 2022 9 18 50.0 [26.0; 74.0]
Liang 2025 20 25 80.0 [59.3; 93.2]
Pooled (events / N, exact CI) 345 32.2 [27.3; 37.4]
Heterogeneity: I^2 = 87.0%, p < 0.0001
0 20 40 60 80
Any adverse event rate (%)
B
Study Events N MUA Rate (%) 95% CI
Zeng 2026 0 35 0.0 [0.0; 10.0]
Sujir 2025 0 50 0.0 [0.0; 7.1]
Lee 2020 0 57 0.0 [0.0; 6.3]
Armstrong 2020 0 115 0.0 [0.0; 3.2]
Kim 2020 0 30 0.0 [0.0; 11.6]
Saied 2016 0 25 0.0 [0.0; 13.7]
Atoun 2013 0 33 0.0 [0.0; 10.6]
Wang 2007 0 51 0.0 [0.0; 7.0]
Ko 2021 1 281 0.4 [0.0; 2.0]
Song 2021 1 141 0.7 [0.0; 3.9]
Inglese 2024 2 110 1.8 [0.2; 6.4]
Liu 2026 4 186 2.2 [0.6; 5.4]
Saito 2017 1 22 4.5 [0.1; 22.8]
Takahashi 2019 4 68 5.9 [1.6; 14.4]
Kraal 2024 3 40 7.5 [1.6; 20.4]
Rangan 2020 16 201 8.0 [4.6; 12.6]
Sundararajan 2022 6 41 14.6 [5.6; 29.2]
Mohanty 2025 3 15 20.0 [4.3; 48.1]
Saito 2023 13 51 25.5 [14.3; 39.6]
Shah 2024 15 35 42.9 [26.3; 60.6]
Pooled (events / N, exact CI) 1587 4.3 [3.4; 5.5]
Heterogeneity: I^2 = 82.9%, p < 0.0001
0 10 20 30 40 50 60
Any adverse event rate (%)
C
Study Events N ACR Rate (%) 95% CI
Zeng 2026 0 31 0.0 [0.0; 11.2]
Ziegenfuss 2024 0 51 0.0 [0.0; 7.0]
Li 2023 0 81 0.0 [0.0; 4.5]
Tawfeek 2022 0 32 0.0 [0.0; 10.9]
Mardani−Kivi 2021 0 51 0.0 [0.0; 7.0]
Hasegawa 2021 0 60 0.0 [0.0; 6.0]
Satora 2021 0 30 0.0 [0.0; 11.6]
Kim 2020 0 30 0.0 [0.0; 11.6]
Gallacher 2018 0 19 0.0 [0.0; 17.6]
Cvetanovich 2018 0 27 0.0 [0.0; 12.8]
Cho 2016 0 37 0.0 [0.0; 9.5]
Mehta 2014 0 42 0.0 [0.0; 8.4]
Sabat 2008 0 20 0.0 [0.0; 16.8]
Saied 2016 0 27 0.0 [0.0; 12.8]
Bidwai 2016 0 54 0.0 [0.0; 6.6]
Dattani 2013 0 100 0.0 [0.0; 3.6]
Chen 2010 0 70 0.0 [0.0; 5.1]
Ranalletta 2017 1 32 3.1 [0.1; 16.2]
Mohanty 2025 1 15 6.7 [0.2; 31.9]
Rangan 2020 21 203 10.3 [6.5; 15.4]
Lee 2020 4 22 18.2 [5.2; 40.3]
Sundararajan 2022 10 44 22.7 [11.5; 37.8]
Pooled (events / N, exact CI) 1078 3.4 [2.4; 4.7]
Heterogeneity: I^2 = 60.1%, p = 0.0002
0 10 20 30 40
Any adverse event rate (%)
