# Supplementary material for: Transarterial Embolization Versus Manipulation Under Anesthesia and Arthroscopic Capsular Release for Refractory Adhesive Capsulitis—A Cost-Effectiveness Analysis
Source: Cardiovasc Intervent Radiol. 2026 Aug 2;49(10):1977–88. doi: 10.1007/s00270-026-04563-8 (PMC13642511; doi:10.1007/s00270-026-04563-8)
Supplement: Supplementary file 12 [file 270_2026_4563_MOESM8_ESM.pdf]

A

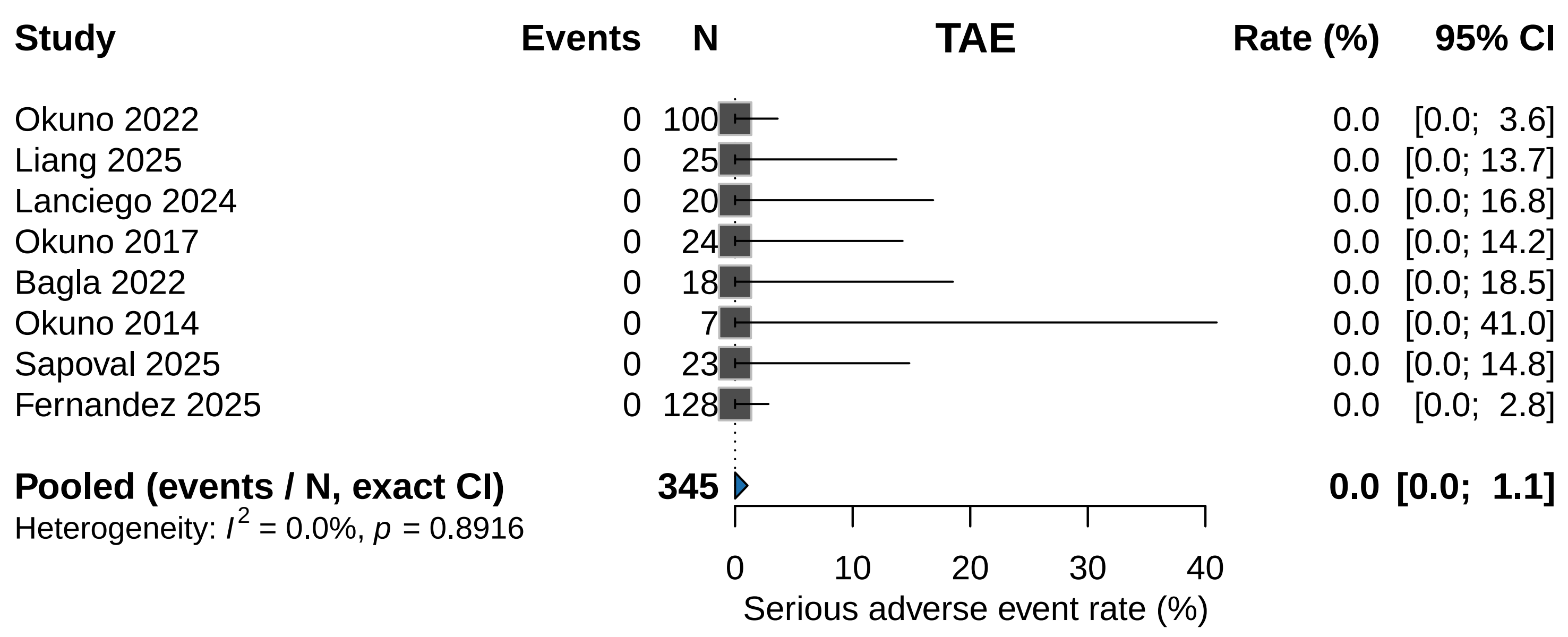
Study
Events
N
TAE
Rate (%)
95% CI
Okuno 2022 0 100 0.0 [0.0; 3.6]
Liang 2025 0 25 0.0 [0.0; 13.7]
Lanciego 2024 0 20 0.0 [0.0; 16.8]
Okuno 2017 0 24 0.0 [0.0; 14.2]
Bagla 2022 0 18 0.0 [0.0; 18.5]
Okuno 2014 0 7 0.0 [0.0; 41.0]
Sapoval 2025 0 23 0.0 [0.0; 14.8]
Fernandez 2025 0 128 0.0 [0.0; 2.8]
Pooled (events / N, exact CI) 345 0.0 [0.0; 1.1]
Heterogeneity: I^2 = 0.0%, p = 0.8916
0
10
20
30
40
Serious adverse event rate (%)

B

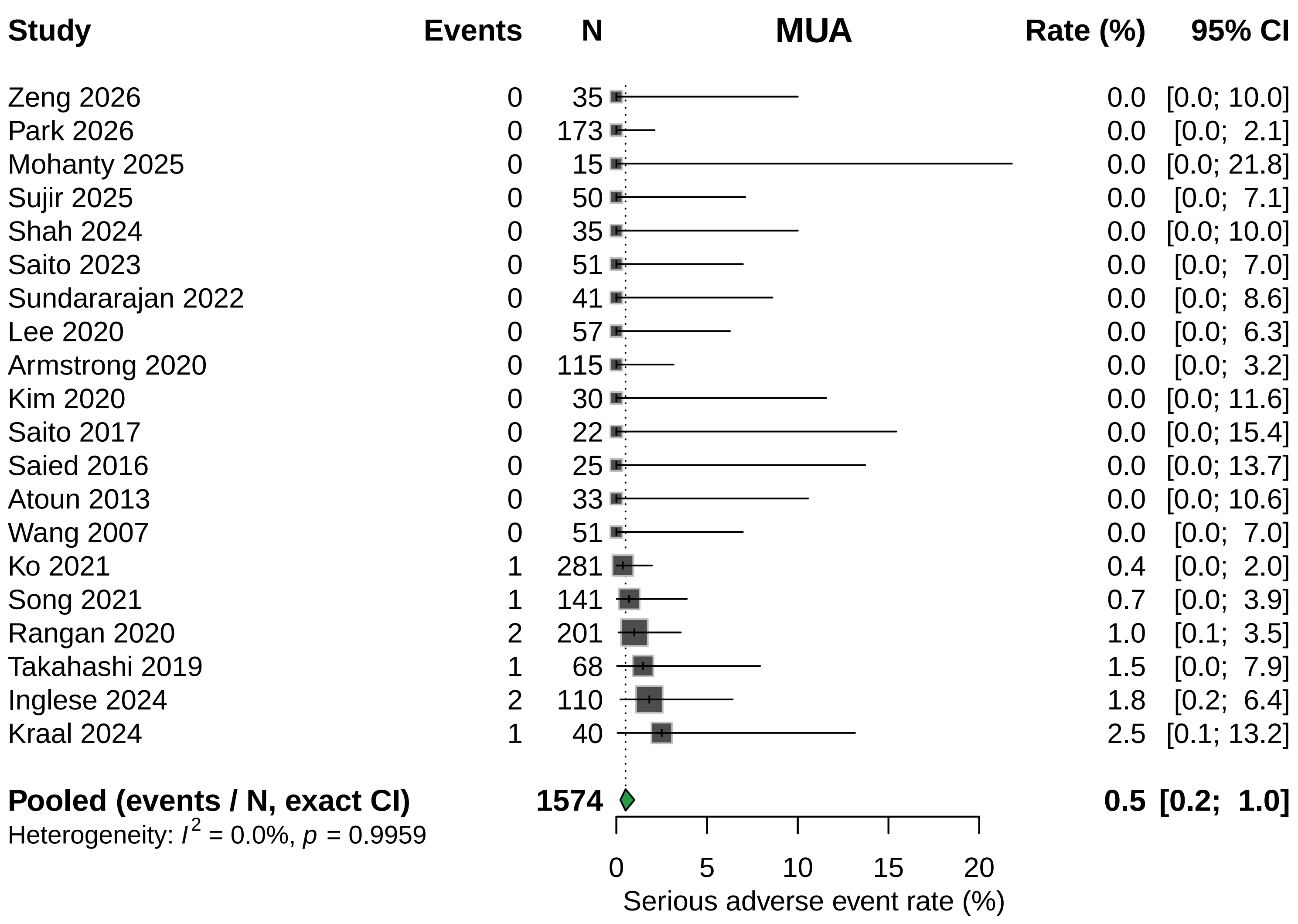
Study
Events
N
MUA
Rate (%)
95% CI
Zeng 2026 0 35 0.0 [0.0; 10.0]
Park 2026 0 173 0.0 [0.0; 2.1]
Mohanty 2025 0 15 0.0 [0.0; 21.8]
Sujir 2025 0 50 0.0 [0.0; 7.1]
Shah 2024 0 35 0.0 [0.0; 10.0]
Saito 2023 0 51 0.0 [0.0; 7.0]
Sundararajan 2022 0 41 0.0 [0.0; 8.6]
Lee 2020 0 57 0.0 [0.0; 6.3]
Armstrong 2020 0 115 0.0 [0.0; 3.2]
Kim 2020 0 30 0.0 [0.0; 11.6]
Saito 2017 0 22 0.0 [0.0; 15.4]
Saied 2016 0 25 0.0 [0.0; 13.7]
Atoun 2013 0 33 0.0 [0.0; 10.6]
Wang 2007 0 51 0.0 [0.0; 7.0]
Ko 2021 1 281 0.4 [0.0; 2.0]
Song 2021 1 141 0.7 [0.0; 3.9]
Rangan 2020 2 201 1.0 [0.1; 3.5]
Takahashi 2019 1 68 1.5 [0.0; 7.9]
Inglese 2024 2 110 1.8 [0.2; 6.4]
Kraal 2024 1 40 2.5 [0.1; 13.2]
Pooled (events / N, exact CI) 1574 0.5 [0.2; 1.0]
Heterogeneity: I^2 = 0.0%, p = 0.9959
0
5
10
15
20
Serious adverse event rate (%)

C

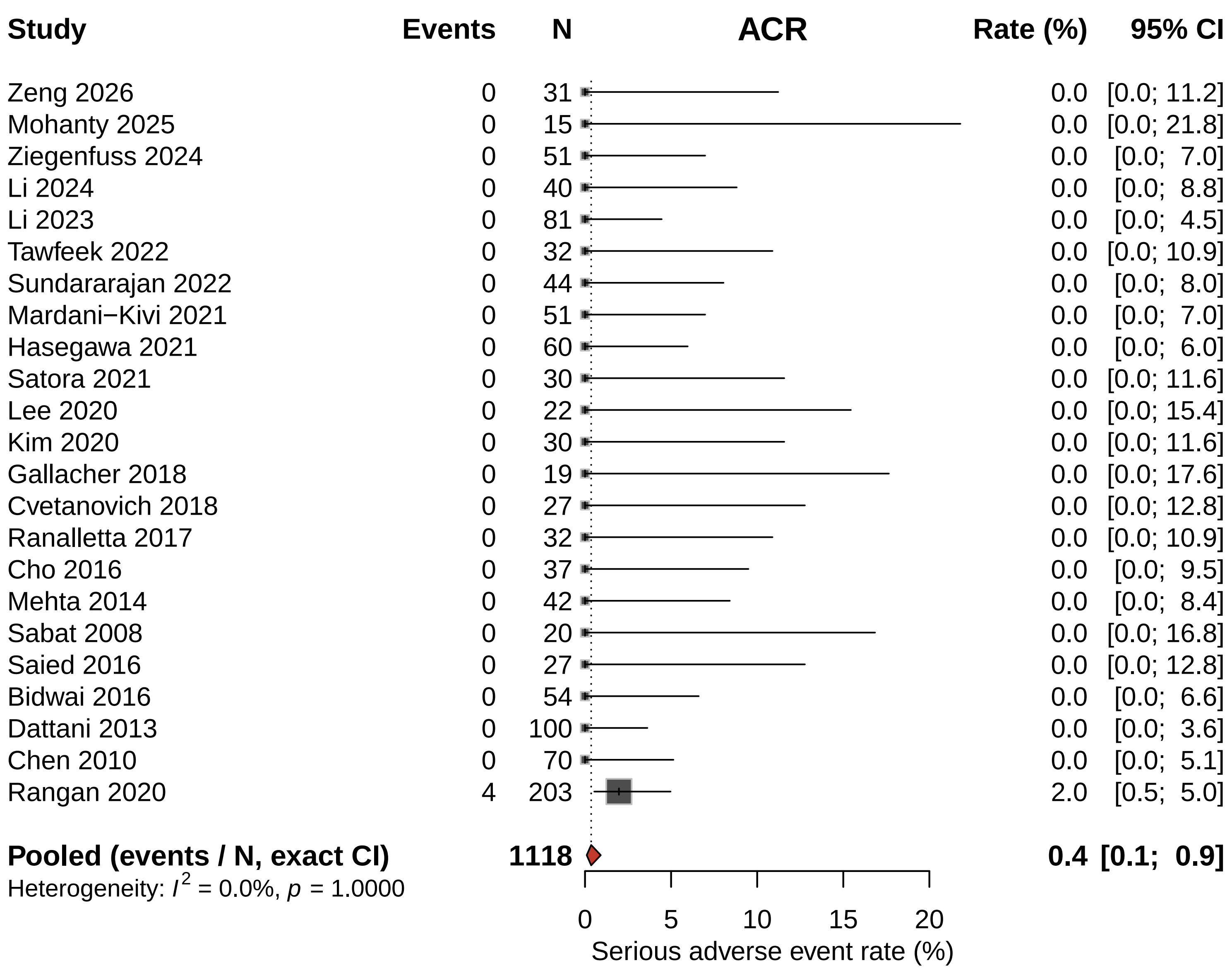
Study
Events
N
ACR
Rate (%)
95% CI
Zeng 2026 0 31 0.0 [0.0; 11.2]
Mohanty 2025 0 15 0.0 [0.0; 21.8]
Ziegenfuss 2024 0 51 0.0 [0.0; 7.0]
Li 2024 0 40 0.0 [0.0; 8.8]
Li 2023 0 81 0.0 [0.0; 4.5]
Tawfeek 2022 0 32 0.0 [0.0; 10.9]
Sundararajan 2022 0 44 0.0 [0.0; 8.0]
Mardani−Kivi 2021 0 51 0.0 [0.0; 7.0]
Hasegawa 2021 0 60 0.0 [0.0; 6.0]
Satora 2021 0 30 0.0 [0.0; 11.6]
Lee 2020 0 22 0.0 [0.0; 15.4]
Kim 2020 0 30 0.0 [0.0; 11.6]
Gallacher 2018 0 19 0.0 [0.0; 17.6]
Cvetanovich 2018 0 27 0.0 [0.0; 12.8]
Ranalletta 2017 0 32 0.0 [0.0; 10.9]
Cho 2016 0 37 0.0 [0.0; 9.5]
Mehta 2014 0 42 0.0 [0.0; 8.4]
Sabat 2008 0 20 0.0 [0.0; 16.8]
Saied 2016 0 27 0.0 [0.0; 12.8]
Bidwai 2016 0 54 0.0 [0.0; 6.6]
Dattani 2013 0 100 0.0 [0.0; 3.6]
Chen 2010 0 70 0.0 [0.0; 5.1]
Rangan 2020 4 203 2.0 [0.5; 5.0]
Pooled (events / N, exact CI) 1118 0.4 [0.1; 0.9]
Heterogeneity: I^2 = 0.0%, p = 1.0000
0
5
10
15
20
Serious adverse event rate (%)
