# Supplementary material for: Transarterial Embolization Versus Manipulation Under Anesthesia and Arthroscopic Capsular Release for Refractory Adhesive Capsulitis—A Cost-Effectiveness Analysis
Source: Cardiovasc Intervent Radiol. 2026 Aug 2;49(10):1977–88. doi: 10.1007/s00270-026-04563-8 (PMC13642511; doi:10.1007/s00270-026-04563-8)
Supplement: Supplementary file 14 [file 270_2026_4563_MOESM17_ESM.docx]

Table S1. Characteristics of included transarterial embolization (TAE) studies. Max follow-up = longest timepoint contributing extracted outcome data. NR = not reported or no poolable outcome extracted for that study.

| **Study** | **Design** | **Country** | **N (patients)** | **Age (y)** | **Sex** | **Max follow-up** |
| --- | --- | --- | --- | --- | --- | --- |
| Bagla 2022 | Prospective | United States | 20 | 50.8 ± 9.4 | 18 F / 2 M | 6mo |
| Fernandez 2025 | Retrospective | Spain | 128 | 52.4 (range 35–76) | 84 F / 34 M | 6mo |
| Lanciego 2024 | Prospective | Spain | 20 | median 50.7 (IQR 45.5–55) | 12 F / 8 M | 6mo |
| Liang 2025 | Retrospective | Taiwan | 25 | 54.9 ± 7.1 | 14 F / 11 M | 6mo |
| Okuno 2014 | Prospective | Japan | 7 | 50.3 ± 10.4 | 5 F / 2 M | 6mo |
| Okuno 2017 | Prospective | Japan | 25 | 53.8 (range 39–68) | 16 F / 9 M | 36mo |
| Okuno 2022 | Prospective | Japan | 100 | 58.7 ± 10.5 | 55 F / 45 M | 6mo |
| Sapoval 2025 | Retrospective | France, Argentina | 23 | 51 ± 10 | 17 F / 6 M | 3mo |
| Shintaku 2023 | Retrospective | Japan | 15 | 54.9 ± 7.18 | 9 F / 6 M | 1mo |
| Wang 2026 | Retrospective | Taiwan | 21 | 56.1 ± 6.3 | 14 F / 7 M | 6mo |
