# Supplementary material for: Transarterial Embolization Versus Manipulation Under Anesthesia and Arthroscopic Capsular Release for Refractory Adhesive Capsulitis—A Cost-Effectiveness Analysis
Source: Cardiovasc Intervent Radiol. 2026 Aug 2;49(10):1977–88. doi: 10.1007/s00270-026-04563-8 (PMC13642511; doi:10.1007/s00270-026-04563-8)
Supplement: Supplementary file 15 [file 270_2026_4563_MOESM18_ESM.docx]

Table S2. Characteristics of included manipulation under anaesthesia (MUA) studies. Max follow-up = longest timepoint contributing extracted outcome data. NR = not reported or no poolable outcome extracted for that study.

| **Study** | **Design** | **Country** | **N (patients)** | **Age (y)** | **Sex** | **Max follow-up** |
| --- | --- | --- | --- | --- | --- | --- |
| Ari 2023 | Retrospective | Turkey | 25 | 59.32 ± 11.11 | 17 F / 8 M | 12mo |
| Armstrong 2020 | Retrospective | United Kingdom | 115 | 54 (range 41–58) | 57 F / 58 M | 1mo |
| Atoun 2013 | Prospective | United Kingdom | 33 | 50.3 (range 42–63) | 18 F / 15 M | NR |
| Inglese 2024 | Retrospective | Italy | 110 | 45 (range 36–55) | 44 F / 66 M | 3mo |
| Kim 2020 | Retrospective | South Korea | 30 | 54.5 ± 7.7 | 21 F / 9 M | 12mo |
| Ko 2021 | Retrospective | South Korea | 281 | 52.9 ± 8.1 | 201 F / 80 M | 3mo |
| Kraal 2024 | RCT | Netherlands | 40 | 51.68 ± 7.93 | 22 F / 18 M | 1mo |
| Lee 2020 | Retrospective | South Korea | 57 | 55.3 ± 8.5 | 33 F / 24 M | 6mo |
| Liu 2026 | Prospective | China | 166 | 54 ± 8 | 122 F / 44 M | 6mo |
| Mahmood 2021 | Prospective | Iraq | 22 | NR | 32 F / 8 M (overall) | 6mo |
| Manda 2025 | Prospective | India | 30 | 52.6 ± 8.5 | 25 F / 35 M (overall) | 3mo |
| Mohanty 2025 | RCT | India | 15 | 45–65 (range) | 16 F / 14 M (overall) | NR |
| Park 2026 | Retrospective | Japan | 169 (173 shoulders) | 53.7 ± 6.9 | 123 F / 46 M | 3mo |
| Rangan 2020 | RCT | United Kingdom | 189 (201 as randomized) | 54.4 ± 7.3 | 121 F / 68 M | 12mo |
| Saied 2016 | Prospective | Iran | 25 | 56.84 ± 8.04 (overall) | 43 F / 9 M (overall) | 24mo |
| Saito 2017 | Prospective | Japan | 25 | 58 (range 37–73) | 13 F / 9 M | 12mo |
| Saito 2023 | Prospective | Japan | 51 | median 57 (IQR 51–63) | 34 F / 17 M | 12mo |
| Shah 2024 | Cross-sectional | Nepal | 35 | 53.46 ± 7.524 | 22 F / 13 M | 6mo |
| Sokk 2012 | Prospective | Estonia | 13 | median 53 (range 38–74) | 8 F / 5 M | 6mo |
| Song 2021 | Retrospective | China | 88 | 53.0 ± 5.5 / 55.1 ± 6.4 | 52 F / 36 M | 1mo |
| Sujir 2025 | Retrospective | India | 50 | 57.32 ± 11.78 | 28 F / 22 M | 12mo |
| Sundararajan 2022 | RCT | India | 41 | 53.3 ± 6.7 | NR | 6mo |
| Takahashi 2019 | Retrospective | Japan | 68 | 52.0 ± 7.5 | 42 F / 26 M | 6mo |
| Wang 2007 | Retrospective | Taiwan | 26 | 53.9 (overall; range 38–79) | 33 F / 16 M (overall) | 84mo |
| Zeng 2026 | Retrospective | China | 35 | 47.63 ± 7.30 | 22 F / 13 M | 6mo |
