# Supplementary material for: Transarterial Embolization Versus Manipulation Under Anesthesia and Arthroscopic Capsular Release for Refractory Adhesive Capsulitis—A Cost-Effectiveness Analysis
Source: Cardiovasc Intervent Radiol. 2026 Aug 2;49(10):1977–88. doi: 10.1007/s00270-026-04563-8 (PMC13642511; doi:10.1007/s00270-026-04563-8)
Supplement: Supplementary file 16 [file 270_2026_4563_MOESM19_ESM.docx]

Table S3. Characteristics of included arthroscopic capsular release (ACR) studies. Max follow-up = longest timepoint contributing extracted outcome data. NR = not reported or no poolable outcome extracted for that study.

| **Study** | **Design** | **Country** | **N (patients)** | **Age (y)** | **Sex** | **Max follow-up** |
| --- | --- | --- | --- | --- | --- | --- |
| Alazabi 2021 | Prospective | Egypt | 18 | 51.11 ± 5.91 | 11 F / 7 M | 6mo |
| Ari 2023 | Retrospective | Turkey | 18 | 53.22 ± 11.51 | 11 F / 7 M | 12mo |
| Barbosa 2019 | Prospective | United Kingdom | 97 | 55 (overall; range 33–77) | 130 F / 80 M (overall) | NR |
| Bidwai 2016 | Prospective | United Kingdom | 54 shoulders in 52 patients | median 50 (range 42–59) | 41 F / 11 M | 6mo |
| Chen 2010 | RCT | China | 70 (41 Group 1 + 29 Group 2; 4 lost from 74) | 56 (range 34–77) | 43 F / 31 M | 24mo |
| Cho 2016 | Retrospective | South Korea | 37 patients (17 diabetic, 20 idiopathic) | 55.6 ± 8.1 | 25 F / 12 M | 48mo |
| Cvetanovich 2018 | Retrospective | United States | 43 identified; 10 excluded (post-traumatic/post-surgical); 33 remained; 27 (81.8%) had min 2-year follow-up | 54.8 ± 7.4 | 21 F / 6 M | 48mo |
| Dattani 2013 | Prospective | United Kingdom | 100 | 54.3 ± 9.66 | 60 F / 40 M | 6mo |
| De Virgilio-Salgado 2023 | Retrospective | United States | 134 | 57.4 ± 8.88 | 100 F / 34 M | 6mo |
| Gallacher 2018 | RCT | United Kingdom | 19 | 52.6 ± 8.1 | 16 F / 3 M | 6mo |
| Haroun 2024 | Prospective | Egypt | 57 | 50.18 ± 7.96 | 35 F / 22 M | 12mo |
| Hasegawa 2021 | Retrospective | Japan | 60 | 60.1 (range 37–78) | 35 F / 25 M | 6mo |
| Kim 2020 | Retrospective | South Korea | 30 | 55.3 ± 8.3 | 21 F / 9 M | 12mo |
| Lee 2020 | Retrospective | South Korea | 22 | 53.9 ± 6.4 | 14 F / 8 M | 6mo |
| Li 2023 | Retrospective | China | 81 | 58.2 ± 7.9 / 59.6 ± 6.8 | 46 F / 35 M | 24mo |
| Li 2024 | Retrospective | China | 40 | 53.35 ± 9.61 | 21 F / 19 M | 6mo |
| Mardani-Kivi 2021 | Retrospective | Iran | 51 | 49.1 ± 5.6 | 19 F / 32 M | 48mo |
| Mehta 2014 | Prospective | United Kingdom | 42 (21 DM / 21 non-DM) | 54.5 | 24 F / 18 M | 24mo |
| Mohanty 2025 | RCT | India | 15 | 45–65 (range) | 16 F / 14 M (overall) | NR |
| Pasqualini 2024a | Retrospective | Argentina | 100 | 54.9 ± 8.8 | 76 F / 24 M | 6mo |
| Pasqualini 2024b | Prospective | Argentina | 73 | 55.52 ± 9.3 | 56 F / 17 M | 12mo |
| Pippal 2022 | Prospective | India | 10 | 54.4 ± 7.4 | 8 F / 2 M | 6mo |
| Ranalletta 2017 | Prospective | Argentina | 32 (from 53 considered; 20 excluded for criteria, 1 lost to FU) | 49.6 (range 33–68) | 22 F / 10 M | 60mo |
| Rangan 2020 | RCT | United Kingdom | 191 (203 randomized) | 54.4 ± 7.6 | 117 F / 74 M | 12mo |
| Sabat 2008 | Prospective | India | 20 | 53.65 (range 37–66) | 8 F / 12 M | 12mo |
| Sadeghifar 2015 | Prospective | Iran | 15 (20 operated; 15 attended follow-up) | 50.57 ± 12.01 | 9 F / 6 M | 12mo |
| Saied 2016 | Prospective | Iran | 27 (ACR arm) | 56.84 ± 8.04 (overall) | 43 F / 9 M (overall) | 24mo |
| Satora 2021 | Retrospective | Poland | 59 | 49.8 ± 2.6 / 49.7 ± 4.7 | 37 F / 22 M | 12mo |
| Sundararajan 2022 | RCT | India | 44 | 54.2 ± 5.9 | NR | 6mo |
| Tan 2009 | RCT | United Kingdom | 30 (Splint: 15; No splint: 15) | 52 (range 40–67) | 22 F / 8 M | 12mo |
| Tawfeek 2022 | Prospective | Egypt | 32 (diabetic n=19; non-diabetic n=13) | 47.5 ± 6.2 / 50.2 ± 5.7 | 26 F / 6 M | 12mo |
| Wang 2026 | Retrospective | Taiwan | 19 | 59.1 ± 7.6 | 15 F / 4 M | 6mo |
| Yanlei 2019 | Retrospective | Singapore | 56 | 56.9 / 53.6 | 24 F / 32 M | NR |
| Yoo 2018 | Retrospective | South Korea | 29 (30 shoulders) | 53.8 ± 10.2 | 17 F / 12 M | 24mo |
| Zeng 2026 | Retrospective | China | 31 | 47.45 ± 7.25 | 21 F / 10 M | 6mo |
| Ziegenfuss 2024 | Prospective | Australia | 51 | 52.1 ± 7.7 | 30 F / 21 M | 24mo |
