# Supplementary material for: Transarterial Embolization Versus Manipulation Under Anesthesia and Arthroscopic Capsular Release for Refractory Adhesive Capsulitis—A Cost-Effectiveness Analysis
Source: Cardiovasc Intervent Radiol. 2026 Aug 2;49(10):1977–88. doi: 10.1007/s00270-026-04563-8 (PMC13642511; doi:10.1007/s00270-026-04563-8)
Supplement: Supplementary file 17 [file 270_2026_4563_MOESM20_ESM.docx]

Table S4. Cost-effectiveness results for success defined by forward elevation ROM improvement across all three practice settings Within each setting, strategies are sorted by total cost; ICERs are computed sequentially along the cost-effectiveness frontier (each non-dominated strategy vs the next-cheaper non-dominated strategy, with incremental cost and QALYs reported against that same comparator). NMB, net monetary benefit at the $100,000/QALY threshold; CE probability, % of 1,000 PSA draws with the highest NMB.

| **Setting** | **Strategy** | **Total cost (USD)** | **QALYs** | **Incremental cost (USD)** | **Incremental QALYs** | **ICER (USD/QALY)** | **NMB (USD)** | **CE probability** |
| --- | --- | --- | --- | --- | --- | --- | --- | --- |
| HOPD | MUA | 1,991,737 | 1,975.4 | 0 | 0.00 | — (reference) | 195,552,897 | 99.6% |
|  | ACR | 4,083,016 | 1,939.5 | 2,091,279 | -35.96 | Dominated | 189,865,709 | 0.4% |
|  | TAE | 19,368,949 | 1,752.0 | 17,377,212 | -223.41 | Dominated | 155,834,413 | 0% |
| ASC | MUA | 1,227,456 | 1,975.4 | 0 | 0.00 | — (reference) | 196,317,179 | 99.7% |
|  | ACR | 2,397,517 | 1,939.5 | 1,170,062 | -35.96 | Dominated | 191,551,208 | 0.3% |
|  | TAE | 12,143,173 | 1,752.0 | 10,915,717 | -223.41 | Dominated | 163,060,189 | 0% |
| OBL | MUA | 1,227,456 | 1,975.4 | 0 | 0.00 | — (reference) | 196,317,179 | 99.5% |
|  | ACR | 2,397,517 | 1,939.5 | 1,170,062 | -35.96 | Dominated | 191,551,208 | 0.5% |
|  | TAE | 8,074,898 | 1,752.0 | 6,847,442 | -223.41 | Dominated | 167,128,464 | 0% |
