# Supplementary material for: Transarterial Embolization Versus Manipulation Under Anesthesia and Arthroscopic Capsular Release for Refractory Adhesive Capsulitis—A Cost-Effectiveness Analysis
Source: Cardiovasc Intervent Radiol. 2026 Aug 2;49(10):1977–88. doi: 10.1007/s00270-026-04563-8 (PMC13642511; doi:10.1007/s00270-026-04563-8)
Supplement: Supplementary file 18 [file 270_2026_4563_MOESM21_ESM.docx]

**Supplemental Methods**

***S1. Search Strategy***

The following databases were searched: PubMed, Scopus, and Web of Science. Full Boolean strings for each database and each arm — transarterial embolization (TAE), manipulation under anesthesia (MUA), and arthroscopic capsular release (ACR) — are reproduced verbatim below.

TAE PubMed: (((shoulder*[tiab] OR "upper extremity*"[tiab] OR "upper limb*"[tiab] OR "rotator cuff*"[tiab] OR subacromial*[tiab] OR scapulohumeral*[tiab] OR supraspinatus*[tiab] OR infraspinatus*[tiab]) AND (pain*[tiab])) OR ((“Rotator Cuff Injuries*”[tiab] OR “Shoulder Impingement Syndrome*”[tiab] OR “Bursitis*”[tiab] OR "Adhesive Capsulitis*"[tiab] OR "Frozen Shoulder*"[tiab] OR "Stiff Shoulder*"[tiab] OR "Periarthritis humeroscapularis"[tiab] OR "Degenerative Joint Disease*"[tiab] OR tendinitis*[tiab] OR tendonitis*[tiab] OR tendinopathy*[tiab] OR "Calcific Tendinitis*"[tiab] OR arthritis*[tiab] OR osteoarthritis*[tiab] OR myalgia*[tiab] OR "Rotator Cuff Tear*"[tiab] ) AND (pain*[tiab]))) AND (emboliz*[tiab] OR embolisation*[tiab] OR "transcatheter arterial emboliz*"[tiab] OR "transcatheter arterial embolisation*"[tiab] OR "transcatheter emboliz*"[tiab] OR "arterial emboliz*"[tiab] OR "arterial embolisation*"[tiab] OR "endovascular emboliz*"[tiab] OR microemboliz*[tiab] OR "embolization therapy"[tiab] OR embolotherapy[tiab] OR "vascular occlusion*"[tiab] OR "transarterial emboliz*"[tiab] OR "superselective arterial emboliz*"[tiab] OR TAE[tiab])

TAE Scopus: (( (TITLE-ABS-KEY (shoulder* OR "upper extremity*" OR "upper limb*" OR "rotator cuff*" OR subacromial* OR scapulohumeral* OR supraspinatus* OR infraspinatus*)) AND TITLE-ABS-KEY(pain*)) OR (TITLE-ABS-KEY ("Rotator Cuff Injuries*" OR "Shoulder Impingement Syndrome*" OR bursitis* OR "Adhesive Capsulitis*" OR "Frozen Shoulder*" OR "Stiff Shoulder*" OR "Periarthritis humeroscapularis" OR "Degenerative Joint Disease*" OR tendinitis* OR tendonitis* OR tendinopathy* OR "Calcific Tendinitis*" OR arthritis* OR osteoarthritis* OR myalgia* OR "Rotator Cuff Tear*") AND TITLE-ABS-KEY(pain*))) AND (TITLE-ABS-KEY (emboliz* OR embolisation* OR "transcatheter arterial emboliz*" OR "transcatheter arterial embolisation*" OR "transcatheter emboliz*" OR "arterial emboliz*" OR "arterial embolisation*" OR "endovascular emboliz*" OR microemboliz* OR "embolization therapy" OR embolotherapy OR "vascular occlusion*" OR "transarterial emboliz*" OR "superselective arterial emboliz*" OR TAE))

TAE Web of Science: (((TS=(shoulder* OR "upper extremity*" OR "upper limb*" OR "rotator cuff*" OR subacromial* OR scapulohumeral* OR supraspinatus* OR infraspinatus*)) AND TS=(pain*)) OR ((TS=("Rotator Cuff Injuries*" OR "Shoulder Impingement Syndrome*" OR Bursitis* OR "Adhesive Capsulitis*" OR "Frozen Shoulder*" OR "Stiff Shoulder*" OR "Periarthritis humeroscapularis" OR "Degenerative Joint Disease*" OR tendinitis* OR tendonitis* OR tendinopathy* OR "Calcific Tendinitis*" OR arthritis* OR osteoarthritis* OR myalgia* OR "Rotator Cuff Tear*")) AND TS=(pain*))) AND TS=(emboliz* OR embolisation* OR "transcatheter arterial emboliz*" OR "transcatheter arterial embolisation*" OR "transcatheter emboliz*" OR "arterial emboliz*" OR "arterial embolisation*" OR "endovascular emboliz*" OR microemboliz* OR "embolization therapy" OR embolotherapy OR "vascular occlusion*" OR "transarterial emboliz*" OR "superselective arterial emboliz*" OR TAE)

MUA PubMed: (shoulder*[tiab] OR "upper extremity*"[tiab] OR "upper limb*"[tiab] OR "rotator cuff*"[tiab] OR subacromial*[tiab] OR scapulohumeral*[tiab] OR supraspinatus*[tiab] OR infraspinatus*[tiab] OR "Rotator Cuff Injuries*"[tiab] OR "Shoulder Impingement Syndrome*"[tiab] OR "Bursitis*"[tiab] OR "Adhesive Capsulitis*"[tiab] OR "Frozen Shoulder*"[tiab] OR "Stiff Shoulder*"[tiab] OR "Periarthritis humeroscapularis"[tiab] OR "Degenerative Joint Disease*"[tiab] OR tendinitis*[tiab] OR tendonitis*[tiab] OR tendinopathy*[tiab] OR "Calcific Tendinitis*"[tiab] OR myalgia*[tiab] OR "Rotator Cuff Tear*"[tiab]) AND (MUA[tiab] OR "manipulation under anesthesia"[tiab] OR "manipulation under anaesthesia"[tiab] OR "manipulation under anesthetic"[tiab] OR "manipulation under anaesthetic"[tiab] OR "closed manipulation"[tiab] OR "manual manipulation"[tiab] OR "shoulder manipulation"[tiab] OR "manipulative procedure*"[tiab])

MUA Scopus: TITLE-ABS-KEY(shoulder* OR "upper extremity*" OR "upper limb*" OR "rotator cuff*" OR subacromial* OR scapulohumeral* OR supraspinatus* OR infraspinatus* OR "Rotator Cuff Injuries*" OR "Shoulder Impingement Syndrome*" OR bursitis* OR "Adhesive Capsulitis*" OR "Frozen Shoulder*" OR "Stiff Shoulder*" OR "Periarthritis humeroscapularis" OR "Degenerative Joint Disease*" OR tendinitis* OR tendonitis* OR tendinopathy* OR "Calcific Tendinitis*" OR myalgia* OR "Rotator Cuff Tear*") AND TITLE-ABS-KEY(MUA OR "manipulation under anesthesia" OR "manipulation under anaesthesia" OR "manipulation under anesthetic" OR "manipulation under anaesthetic" OR "closed manipulation" OR "manual manipulation" OR "shoulder manipulation" OR "manipulative procedure")

MUA Web of Science: TS=(shoulder* OR "upper extremity*" OR "upper limb*" OR "rotator cuff*" OR subacromial* OR scapulohumeral* OR supraspinatus* OR infraspinatus* OR "Rotator Cuff Injuries*" OR "Shoulder Impingement Syndrome*" OR Bursitis* OR "Adhesive Capsulitis*" OR "Frozen Shoulder*" OR "Stiff Shoulder*" OR "Periarthritis humeroscapularis" OR "Degenerative Joint Disease*" OR tendinitis* OR tendonitis* OR tendinopathy* OR "Calcific Tendinitis*" OR myalgia* OR "Rotator Cuff Tear*") AND TS=(MUA OR "manipulation under anesthesia" OR "manipulation under anaesthesia" OR "manipulation under anesthetic" OR "manipulation under anaesthetic" OR "closed manipulation" OR "manual manipulation" OR "shoulder manipulation" OR "manipulative procedure*")

ACR PubMed: (shoulder*[tiab] OR "upper extremity*"[tiab] OR "upper limb*"[tiab] OR "rotator cuff*"[tiab] OR subacromial*[tiab] OR scapulohumeral*[tiab] OR supraspinatus*[tiab] OR infraspinatus*[tiab] OR "Rotator Cuff Injuries*"[tiab] OR "Shoulder Impingement Syndrome*"[tiab] OR "Bursitis*"[tiab] OR "Adhesive Capsulitis*"[tiab] OR "Frozen Shoulder*"[tiab] OR "Stiff Shoulder*"[tiab] OR "Periarthritis humeroscapularis"[tiab] OR "Degenerative Joint Disease*"[tiab] OR tendinitis*[tiab] OR tendonitis*[tiab] OR tendinopathy*[tiab] OR "Calcific Tendinitis*"[tiab] OR myalgia*[tiab] OR "Rotator Cuff Tear*"[tiab]) AND ("arthroscopic capsular release"[tiab] OR "capsular release"[tiab] OR "arthroscopic release"[tiab] OR "arthroscopic capsulotomy"[tiab] OR "arthroscopic lysis"[tiab])

ACR Scopus: TITLE-ABS-KEY(shoulder* OR "upper extremity*" OR "upper limb*" OR "rotator cuff*" OR subacromial* OR scapulohumeral* OR supraspinatus* OR infraspinatus* OR "Rotator Cuff Injuries*" OR "Shoulder Impingement Syndrome*" OR bursitis* OR "Adhesive Capsulitis*" OR "Frozen Shoulder*" OR "Stiff Shoulder*" OR "Periarthritis humeroscapularis" OR "Degenerative Joint Disease*" OR tendinitis* OR tendonitis* OR tendinopathy* OR "Calcific Tendinitis*" OR myalgia* OR "Rotator Cuff Tear*") AND TITLE-ABS-KEY("arthroscopic capsular release" OR "capsular release" OR "arthroscopic release" OR "arthroscopic capsulotomy" OR "arthroscopic lysis")

ACR Web of Science: TS=(shoulder* OR "upper extremity*" OR "upper limb*" OR "rotator cuff*" OR subacromial* OR scapulohumeral* OR supraspinatus* OR infraspinatus* OR "Rotator Cuff Injuries*" OR "Shoulder Impingement Syndrome*" OR Bursitis* OR "Adhesive Capsulitis*" OR "Frozen Shoulder*" OR "Stiff Shoulder*" OR "Periarthritis humeroscapularis" OR "Degenerative Joint Disease*" OR tendinitis* OR tendonitis* OR tendinopathy* OR "Calcific Tendinitis*" OR myalgia* OR "Rotator Cuff Tear*") AND TS=("arthroscopic capsular release" OR "capsular release" OR "arthroscopic release" OR "arthroscopic capsulotomy" OR "arthroscopic lysis")

***S2. Study Selection***

Study selection proceeded in two stages. In the first stage, records retrieved from the database searches were deduplicated. Two reviewers (KK and RA) independently screened all titles and abstracts identified by the search strategy against the eligibility criteria below; disagreements at this stage were resolved in favor of inclusion to ensure no potentially eligible study was missed. In the second stage, the same two reviewers independently assessed the full text of all retained records; disagreements were resolved by consensus adjudication with a third reviewer (LA). When multiple publications reported overlapping patient cohorts, the largest or most recent publication was retained.

Quality appraisal of each included study was performed using validated risk-of-bias instruments: the Cochrane Risk-of-Bias 2 (RoB 2) tool was used for randomized controlled trials, and the Risk of Bias in Non-Randomized Studies of Interventions (ROBINS-I) tool was used for non-randomized studies. Each study was assessed independently by two reviewers (KK and RA), with discrepancies resolved by a third reviewer (LA). Per-domain and overall risk-of-bias judgments are reported in traffic-light plots (Supplementary Figures S2 and S3) and domain-level summary proportions (Figure S1). Studies were not excluded based on their risk-of-bias rating. Instead, risk-of-bias findings were incorporated into the interpretation of results through narrative discussion, contour-enhanced funnel plots with Egger's regression test (where ≥10 studies were available), and a leave-one-TAE-trial-out sensitivity analysis.^1^

The eligibility criteria are as follows. We included English-language, peer-reviewed studies of human adults (≥18 years) with primary adhesive capsulitis (AC), defined as gradual onset of shoulder pain with restriction of both active and passive glenohumeral motion in the absence of a precipitating traumatic, surgical, or neurologic cause. Patients with recognized risk factors for primary AC (e.g., diabetes mellitus, thyroid disease) were classified as primary and included. Cohorts mixed with other shoulder pathology (e.g. secondary adhesive capsulitis, tendinobursitis) were eligible if primary AC was the dominant indication (>50% of patients). All included studies documented refractoriness to conservative care before the index intervention.

Eligible interventions were TAE, MUA, and ACR performed specifically for AC. TAE was defined as superselective transarterial embolization of shoulder capsular vessels using any embolic agent, performed under local anesthesia with or without conscious sedation. MUA was defined as manipulation of the shoulder under general or regional anesthesia; adjuvant intra-articular injection (steroid or local anesthetic) at the time of MUA was permitted, but MUA combined with hydrodistention or hydrodilation was excluded. ACR was defined as arthroscopic or mini-open capsular release (anterior, posterior, or 360°) as the primary intervention for capsular contracture; concomitant manipulation under anesthesia performed in any sequence (pre- or post-release) was permitted when it served to assess range of motion, guide the extent of release, or confirm adequate release. Adjunctive procedures addressing coexisting pathology (subacromial decompression, acromioplasty, biceps tenotomy) were permitted. Standard peri-procedural regional anesthesia (≤48 hours) was permitted; papers with prolonged postoperative nerve block or catheter-based analgesia (>48 hours) were excluded.

Studies were required to report pre- and post-intervention data for at least one outcome of interest using a validated instrument, at a time point mappable to 1, 3, 6, or 12 months (±30 days), with a minimum of 5 shoulders per intervention arm and sufficient data to compute change from baseline. The following study types were excluded: reviews, editorials, commentaries, conference abstracts without full publication, protocols, animal studies, and non-English publications.

**S3. Data Extraction and Harmonization**

Data from each included study was extracted and independently verified using a structured spreadsheet template. Variables included first author, publication year, country, study design, total enrolment per arm, intervention details, baseline patient characteristics (age, sex, diabetes mellitus, duration of symptoms), pre- and post-intervention means and standard deviations (SDs) for every reported outcome instrument, sample size at each follow-up timepoint, adverse events with grading where reported, and subsequent surgical or procedural retreatment.

The primary clinical outcome was the 3-month change in pain from baseline measured on the Visual Analog Scale (VAS) or Numeric Rating Scale (NRS), harmonized to 0–10. Secondary outcomes were shoulder function (American Shoulder and Elbow Surgeons [ASES] score, Constant–Murley score, Oxford Shoulder Score [OSS], Shoulder Pain and Disability Index [SPADI], Quick Disabilities of the Arm, Shoulder and Hand [QuickDASH]), range of motion (forward elevation, abduction, external and internal rotation, in degrees), EQ-5D health utility index, and serious adverse events (SAEs). EQ-5D is a validated, preference-based instrument that captures health-related quality of life across five dimensions (mobility, self-care, usual activities, pain/discomfort, and anxiety/depression) and generates a single index score anchored at 0 (dead) and 1 (full health).^2^

SAEs were defined according to the UK Frozen Shoulder Trial (UK FroST) as any medical occurrence resulting in threat to life, hospitalization, significant disability or incapacity, a congenital abnormality, or any other medical condition that may require medical or surgical intervention to prevent any of these from occurring.^3^

We applied four harmonization steps before pooling. First, we negated lower-is-better outcome scores so that a positive mean difference always denotes improvement. Second, we mapped all pain instruments (VAS, NRS) to a common 0–10 scale. While most functional and ROM outcomes all had the same scale, the following scores had to be linearly rescaled: SPADI to 0–100, lower is better; OSS to 0–48, higher is better; lumbar internal rotation to the 1–18 vertebral-level scale. When a study reported multiple pain subscores, we selected one per study using a fixed precedence order (overall > unspecified > motion > rest > nocturnal). When a study reported both active and passive range of motion at the same timepoint, the passive measurement was retained for pooling; for external rotation, the at-side measurement was retained when reported alongside abducted external rotation. Third, we mapped each reported follow-up to a bucket using tolerance windows that widen with follow-up duration: 1 month (center 30 days, ±30 days), 3 month (center 90 days, ±30 days), 6 mo (center 180 days, ±60 days), 12 month (center 365 days, ±120 days), and additional long-term buckets (24, 36, 48, 60, 84 months). A row was assigned to the bucket whose center was within tolerance and nearest its reported timepoint (ties resolved to the earlier bucket); if a study contributed more than one row to the same bucket, we retained the row closest to the bucket center. Observations falling outside every tolerance window were excluded from pooling.

Adverse events were extracted as per-arm event counts with denominators, separately from the continuous outcomes, and pooled as the aggregate proportion with a Clopper-Pearson binomial 95% confidence interval. Subsequent surgical or procedural retreatment was extracted as events with cumulative patient-time at risk and used to derive per-quarter attrition rates for the Markov model. Missing SDs were imputed from confidence intervals (z = 1.96), interquartile ranges, or ranges.^4,5^ Data reporting only point estimates without a measure of dispersion were excluded from the analysis. The within-person pre-post correlation r required for the change-score variance was fixed at 0.5.^6^

**S4. Cost-Effectiveness Analysis (CEA) Markov Model**

We constructed a three-state Markov model (Symptomatic, Healthy, absorbing Diseased) with a quarterly (3-month) cycle length and a 36-month time horizon. This horizon was chosen to mirror AC’s natural history, with a 12-month time horizon added as a sensitivity analysis given limited long-term attrition data.^7,8^ The model adopts a U.S. Medicare payer perspective with a 3% annual discount rate applied to costs and quality-adjusted life-years (QALYs) at the start of each quarterly cycle.^7,8^

The model comprises three health states: Symptomatic, Healthy, and Diseased. All patients enter in the Symptomatic state, representing refractory AC at the time of the index procedure. In the first cycle, each Symptomatic patient transitions to the Healthy state (clinical success achieved) with a probability equal to that arm's clinical success rate; the remainder stay Symptomatic. In every subsequent cycle, Symptomatic patients remain Symptomatic: because the modelled cohort has, by inclusion, already failed conservative management. In each cycle, a Healthy patient either remains Healthy or transitions to the Diseased state at that arm's quarterly attrition rate, defined as the pooled subsequent-procedure rate. The Diseased state is absorbing as it represents durable loss of the initial clinical benefit; additionally, the success and durability of re-interventions are not characterized in the available single-arm evidence. No re-intervention cost was assigned to the Diseased state. Although the transition probability from Healthy to Diseased was derived from pooled subsequent-procedure rates, the type of subsequent procedure was heterogeneous and inconsistently reported across studies, precluding reliable assignment of a procedure-specific cost. Patients therefore accrue costs and QALYs according to the time spent in each state over the 36-month (12-cycle) horizon.

Clinical success was defined as a post-treatment pain NRS ≤ 3 at 3 months, anchored to a reference baseline of NRS 7. This follows the clinical success definition and mean pre-treatment scores in the UK FroST.^9^ For each arm, the expected post-treatment NRS was obtained by subtracting the arm's random-effects pooled 3-month MD from the reference baseline of 7: $Y_{post}=7-MD$. The residual patient-level dispersion around that expected score ($s_{post}$) was summarized as an n-weighted mean of the reported study-level post-treatment SD at 3 months. The probability that a randomly selected post-treatment patient achieved success was then evaluated as the standard normal cumulative distribution function (CDF) of the standardized distance between the responder threshold and the expected post-treatment score $p_{success}=\Phi\left( \frac{3-Y_{post}}{s_{post}} \right)$. This anchored absolute-threshold formulation was chosen over a relative threshold due to significant variability in baseline pain levels between arms (7.48 ± 1.61 for TAE, 6.16 ± 1.62 for MUA, and 7.56 ± 1.60 for ACR), following prior evidence synthesis frameworks that recommend constructing the baseline natural history model independently from the treatment effect model, so that the latter is not influenced by assumptions about the baseline.^10^ The NRS ≤ 3 threshold was preferred over minimal clinically important difference (MCID) or Patient Acceptable Symptom State (PASS)-based success definitions because these values vary substantially by treatment, etiology, outcome metric, and derivation method, and are less well established for shoulder pain interventions than for other musculoskeletal conditions. Borrowing MCID or PASS thresholds from other treatment contexts would introduce assumptions that may not generalize to the interventions studied here.

A parallel anchored scenario was specified to define success as forward shoulder elevation of ≥ 120° at 3 months, the threshold below which patients lose the ability to perform most overhead activities of daily living.^11^ Because there is no single pre-treatment baseline for forward elevation (unlike the UK FroST anchor for pain), the reference baseline for this scenario was derived from the data as the n-weighted mean of the reported pre-treatment forward-elevation means across all primary-analysis arms at 3 months (≈ 89°). The expected post-treatment forward elevation for each arm was the reference baseline plus the arm's random-effects pooled 3-month mean difference, and the residual dispersion was the same n-weighted mean of study-level post-treatment SDs. The success probability for each arm was analogously defined as the standard normal CDF of the standardized distance between the expected post-treatment forward elevation and the 120° threshold.

The attrition rate was defined as the per-quarter subsequent-procedure (e.g. MUA, ACR, TAE) rate pooled from primary-review data (events ÷ patient-quarters at risk, follow-up ≥ 90 days); non-surgical retreatment, such as steroid injection, was excluded. Health state utilities were obtained from the EQ-5D health utility index. The Healthy state utility was pooled from reported 6-month post-treatment EQ-5D index scores using an n-weighted mean, with 6 months was selected as the earliest timepoint at which data were available across arms and at which post-procedural recovery is expected to have stabilized. The Symptomatic and Diseased states share the pooled pre-treatment EQ-5D value. All three treatment arms use the same shared per-state utility values based off the assumption that the quality-of-life benefit of treatment success is equivalent regardless of the intervention used. Per-arm SAE probabilities are taken from the pooled meta-analyses (TAE 0% [0/345], MUA 0.5% [8/1574], ACR 0.4% [4/1118]) and applied as a one-time cycle-1 risk. A $3,000 per-event management cost is applied uniformly across arms.

**S5. CEA Cost Inputs**

Procedural costs reflected 2026 Centers for Medicare and Medicaid Services (CMS) national fee schedules. The non-facility and facility prices for each Current Procedural Terminology (CPT) code was derived from the Physician Fee Schedule (PFS) national payment file. The hospital outpatient (HOPD) facility payment per CPT was derived from the Outpatient Prospective Payment System (OPPS) Addendum B, while the ambulatory surgery center (ASC) facility payment per CPT code was derived from the ASC Addendum AA. The CMS anesthesia conversion factor file was used to obtain the 2026 national anesthesia conversion factor. The primary procedure CPT code was 37242 (vascular embolization or occlusion, organic process) for TAE, 23700 (manipulation of shoulder joint requiring anesthesia) for MUA, and 29825 (arthroscopic shoulder capsulotomy with capsular release) for ACR. TAE additionally bills CPT codes 36216 (selective catheter placement, second order), 36218 (selective catheter placement, third order) and CPT 75710 (diagnostic extremity angiography).

For each treatment arm and care setting, we built the per-case Medicare allowable as a sum of separately billable components: the relevant facility payment, the professional fees for the primary and ancillary CPT codes, and anesthesia where applicable. The HOPD and ASC settings bill the facility payment for the primary CPT code, the facility-price professional fee for every billable CPT code, and (for MUA and ACR) the anesthesia line. The OBL cost, which is only computed for TAE (MUA and ACR are not performed in an OBL setting), used the PFS Non-Facility Price for every billable CPT code to construct a single global fee.

General anesthesia for MUA and ACR used CPT 01630 (anesthesia for shoulder procedure). The per-case anesthesia payment is Anesthesia = (base units + time units) × conversion factor where base units = 5 for CPT 01630, time units are billed in 15-minute increments, and the conversion factor was $20.49754. MUA was assumed to take approximately 45 minutes (3 time units, 8 anesthesia units in total), and ACR was assumed to take approximately 75 minutes (5 time units, 10 anesthesia units in total). TAE is performed under local anesthesia with conscious sedation and therefore carried no anesthesia cost in the model.

**S6. CEA Probabilistic and Deterministic Sensitivity Analyses**

For the probabilistic sensitivity analysis (PSA), we ran 1,000 Monte Carlo iterations per scenario with a fixed random seed. Parameters representing probabilities were sampled from Beta(α, β) distributions with α = μ × n_eff and β = (1 − μ) × n_eff, where n_eff is the effective evidence sample size (i.e. post-treatment N for success, patient-quarters at risk for attrition, total N for SAE). Utilities used Beta priors with n_eff equal to the total weighted N pooled from the EQ-5D evidence base. Note that adverse-event probabilities used a Laplace-corrected Beta(events+1, N−events+1) so the zero-event TAE arm is non-degenerate. Parameters representing costs were sampled from Gamma(shape, rate) priors with shape = (1/CV)² and rate = 1/(μ·CV²), with a coefficient of variation CV = 0.25.^12^ Cost-effectiveness planes (TAE vs MUA, ACR vs MUA), cost-effectiveness acceptability curves (CEACs), and summary tables of dominated, dominant, cost-effective, and CE-probability outcomes, and net monetary benefit (NMB) distributions at a willingness-to-pay (WTP) threshold of $100,000/QALY were produced.

Deterministic sensitivity analyses (DSA) included the following: (i) a practice-setting grid (all-ASC base case, all-HOPD, and TAE in OBL with MUA and ACR at ASC); (ii) two-way parameter heatmaps over (success × utility), (attrition × utility), and (success × attrition); and (iii) a leave-one-TAE-trial-out analysis that sequentially re-pools the TAE evidence base and re-runs the full Markov model excluding each TAE study, bounding the influence of any single TAE cohort on the overall results.

**Supplemental Figures**

Figure S1. Risk of bias summary across all included studies. Panel A: overall RoB 2 judgements across the RCTs, stacked as the proportion of studies judged at Low, Some concerns, or High risk for each of the five RoB 2 domains. Panel B: overall ROBINS-I judgments across the 55 non-randomized studies, stacked as the proportion judged at Low, Moderate, Serious, or Critical risk for each of the seven ROBINS-I domains. Per-study domain-level traffic-light assessments are provided in Supplemental Figures S2 and S3.

Figure S2. Per-study RoB 2 risk-of-bias assessment for randomized controlled trials. Traffic-light plot showing domain-level judgments (low, some concerns, or high risk) across the five RoB 2 domains for each of the 7 included RCTs, with an overall judgment per study. Domain-level summary proportions are shown in Figure S1A.

Figure S3. Per-study ROBINS-I risk-of-bias assessment for non-randomized studies. Traffic-light plot showing domain-level judgments (low, moderate, serious, or critical risk) across the seven ROBINS-I domains for each of the 55 included non-randomized studies, with an overall judgment per study. Domain-level summary proportions are shown in Figure S1B.

Figure S4. Single-arm random-effects forest plots of the American Shoulder and Elbow Surgeons (ASES) functional score at 3 months. Per-arm pools combine study-level mean differences in ASES units (0-100, higher = better shoulder function). The diamond at the bottom of each panel is the random-effects pooled mean difference and the dashed horizontal band is its prediction interval. (A) TAE showed a mean ASES improvement of 45.14 (95% CI 21.57-68.71), while MUA showed an average ASES improvement of 42.81 (29.39-56.24), and ACR showed an average ASES improvement of 36.98 (24.61-49.34).

Figure S5. Single-arm random-effects forest plots of external rotation ROM at 3 months. Per-arm pools combine study-level mean differences in external rotation ROM (in degrees). The diamond at the bottom of each panel is the random-effects pooled mean difference and the dashed horizontal band is its prediction interval. (A) TAE showed a mean external rotation ROM improvement of 24.31° (95% CI 16.19°-32.42°), while MUA showed an average improvement of 42.32° (95% CI 30.03°-54.61°), and ACR showed an average improvement of 34.95° (95% CI 25.43°-44.46°).

Figure S6. Outcome trajectories over time. Absolute pooled outcome scores (n-weighted means) for each arm (TAE, MUA, or ACR) are shown at baseline and at 1, 3, 6, and 12 months; because the arms differ at baseline, these absolute levels are distinct from the change-from-baseline mean differences shown in the forest plots (Figures 2, 3, and S4). Panels: (A) pain (VAS/NRS, 0–10; higher = worse), (B) forward elevation and (C) external rotation ROM (degrees), and (D) ASES, (E) Constant–Murley, and (F) OSS (function scales, higher = better).

Figure S7. Single-arm forest plots of any adverse event by treatment arm. Each row is a study's observed event proportion with a Clopper-Pearson 95% CI; the colored diamond is the pooled estimate, reported as the aggregate proportion (total events ÷ total N) with its exact CI. (A) TAE 32.2% (95% CI 27.3–37.4%; 111/345), (B) MUA 4.3% (3.4–5.5%; 69/1587), and (C) ACR 3.4% (2.4–4.7%; 37/1078).

Figure S8. Single-arm forest plots of serious adverse events by treatment arm. Plot elements as in Figure S7; the pooled diamond is the aggregate proportion with a Clopper-Pearson exact 95% CI. (A) TAE 0% (95% CI 0.0–1.1%; 0/345), (B) MUA 0.5% (0.2–1.0%; 8/1574), and (C) ACR 0.4% (0.1–0.9%; 4/1118). No serious adverse event was reported across the eight TAE studies reporting adverse event data.

Figure S9. Contour-enhanced funnel plot for pain at 3 months following TAE (k = 9 studies). Each circle is one study; the horizontal axis is the study-level MD (NRS/VAS units) and the vertical axis is its standard error, with more precise studies toward the top. The dashed inverted triangle is the pseudo-95% confidence region centered on the random-effects pooled estimate (vertical dashed line); shaded bands denote conventional significance regions (p < 0.10, < 0.05, < 0.01). Because pre-to-post effects are uniformly large, all fall within the significant region. With only 8 studies, formal asymmetry testing was not performed.

Figure S10. Contour-enhanced funnel plot for pain at 3 months following MUA (k = 12). Plot elements as in Figure S9. The plot was symmetric, with no evidence of small-study effects (Egger p = 0.96).

Figure S11. Contour-enhanced funnel plot for pain at 3 months following ACR (k = 14). Plot elements as in Figure S9. Egger's test was non-significant (p = 0.41).

Figure S12. Contour-enhanced funnel plot for forward-elevation ROM at 3 months following MUA (k = 8); horizontal axis is the per-study mean difference in degrees. Plot elements as in Figure S9. With 8 studies, formal asymmetry testing was not feasible.

Figure S13. Contour-enhanced funnel plot for forward-elevation ROM at 3 months following ACR (k = 12). Plot elements as in Figure S9. The plot was symmetric (Egger p = 0.86).

Figure S14. Multiway DSA for pain for TAE versus ACR. Heatmaps of the net monetary benefit (NMB) ratio (TAE NMB / ACR NMB) at the $100,000 per QALY willingness-to-pay threshold, swept across three pairs of TAE-specific parameters: (A) TAE healthy-state utility versus TAE cycle-1 clinical success probability, (B) TAE healthy-state utility versus TAE quarterly attrition rate, and (C) TAE quarterly attrition rate versus TAE cycle-1 clinical success probability. Each parameter is varied over the deterministic-sensitivity range; remaining parameters are held at their base-case values. The black contour line marks the NMB ratio of 1.0 (TAE and ACR break-even); blue regions favor TAE (NMB ratio > 1) and red regions favor ACR (NMB ratio < 1). The visual area above the contour line in each panel approximates the joint parameter region in which TAE is preferred to ACR at this WTP.

Figure S15. Leave-one-TAE-trial-out sensitivity analysis of the cost-effectiveness acceptability curves at the 36-month horizon, with clinical success defined as pain NRS ≤ 3 at 3 months. Because the TAE arm rests on nine single-arm pain pools, each TAE study was excluded in turn, the remaining eight were re-pooled, and the cost-effectiveness analysis was re-run with all other parameters held at base-case values. Solid lines are the all-arms-in acceptability curves from the primary analysis; shaded bands are the minimum-to-maximum range across the nine refits, shown for all three strategies because the probability of being cost-effective is a three-way competition that must sum to 100%. The dashed vertical line marks the $100,000/QALY willingness-to-pay threshold. (A) In the HOPD setting, ACR was optimal in the base case, and TAE's probability of being cost-effective ranged from 12.7% (excluding Bagla 2022) to 51.3% (excluding Okuno 2022), overtaking ACR in only 2 of 9 refits.^13,14^ (B) In the ASC setting, TAE was optimal in the base case (73.3%) and remained so in 8 of 9 refits, with its probability ranging from 44.3% to 89.0%. The exception was exclusion of Bagla 2022, after which ACR became optimal (45.6% versus 44.3%).^13^ (C) With TAE in the OBL setting, TAE remained optimal in all 9 refits, with its probability ranging from 84.1% (excluding Bagla 2022) to 99.9% (excluding Okuno 2022) against 96.9% in the base case.^13,14^

Figure S16. Cost-effectiveness frontier at the 12-month horizon with success defined as pain NRS ≤ 3 at 3 months. For each panel, the three diamonds are the treatment strategies plotted by total cohort cost (vertical axis, thousands USD per 1,000-patient cohort) and total QALYs (horizontal axis, thousands per cohort). The light scatter clouds are the 1,000-iteration PSA draws with sampled parameters. The black line is the cost-effectiveness frontier; the gray dashed line is the WTP of $100,000 per QALY drawn through the cheapest strategy. While the 12-month horizon model minimizes extrapolation of long-term treatment durability, a shorter time horizon results in decreased time for TAE’s greater pain reduction to accumulate QALYs (4 cycles vs. 12 cycles in the 36-month horizon model mirroring AC’s natural history). This results in TAE’s cost per QALY to exceed the $100,000/QALY WTP in the (A) HOPD, (B) ASC, and (C) OBL settings, indicating that TAE is not cost-effective when limiting patient outcomes to 12 months after treatment.

**References**

1.	Higgins JPT, Thomas J, Chandler J, Cumpston M, Li T, Page MJ. *Cochrane Handbook for Systematic Reviews of Interventions*. 2nd ed. John Wiley & Sons; 2019. doi:10.1002/9781119536604

2.	Rabin R, Charro F de. EQ-SD: a measure of health status from the EuroQol Group. *Annals of Medicine*. 2001;33(5):337-343. doi:10.3109/07853890109002087

3.	Brealey S, Northgraves M, Kottam L, et al. Surgical treatments compared with early structured physiotherapy in secondary care for adults with primary frozen shoulder: the UK FROST three-arm RCT. *Health Technology Assessment*. 2020;24(71):1-162. doi:10.3310/hta24710

4.	Luo D, Wan X, Liu J, Tong T. Optimally estimating the sample mean from the sample size, median, mid-range, and/or mid-quartile range. *Stat Methods Med Res*. 2018;27(6):1785-1805. doi:10.1177/0962280216669183

5.	Wan X, Wang W, Liu J, Tong T. Estimating the sample mean and standard deviation from the sample size, median, range and/or interquartile range. *BMC Med Res Methodol*. 2014;14(1):135. doi:10.1186/1471-2288-14-135

6.	Morris SB, DeShon RP. Combining effect size estimates in meta-analysis with repeated measures and independent-groups designs. *Psychological Methods*. 2002;7(1):105-125. doi:10.1037/1082-989X.7.1.105

7.	Reeves B. The Natural History of the Frozen Shoulder Syndrome. *Scandinavian Journal of Rheumatology*. 1975;4(4):193-196. doi:10.3109/03009747509165255

8.	Sanders GD, Neumann PJ, Basu A, et al. Recommendations for Conduct, Methodological Practices, and Reporting of Cost-effectiveness Analyses: Second Panel on Cost-Effectiveness in Health and Medicine. *JAMA*. 2016;316(10):1093. doi:10.1001/jama.2016.12195

9.	Rangan A, Brealey SD, Keding A, et al. Management of adults with primary frozen shoulder in secondary care (UK FROST): a multicentre, pragmatic, three-arm, superiority randomised clinical trial. *The Lancet*. 2020;396(10256):977-989. doi:10.1016/S0140-6736(20)31965-6

10.	Dias S, Welton NJ, Sutton AJ, Ades AE. Evidence Synthesis for Decision Making 5: The Baseline Natural History Model. *Med Decis Making*. 2013;33(5):657-670. doi:10.1177/0272989X13485155

11.	Namdari S, Yagnik G, Ebaugh DD, et al. Defining functional shoulder range of motion for activities of daily living. *Journal of Shoulder and Elbow Surgery*. 2012;21(9):1177-1183. doi:10.1016/j.jse.2011.07.032

12.	Briggs A, Claxton K, Sculpher and M. *Decision Modelling for Health Economic Evaluation*. Oxford University Press; 2006.

13.	Bagla S, Nagda S, Piechowiak R, Orlando J, Sajan A, Isaacson A. Results from a United States Investigational Device Study of Adhesive Capsulitis Embolization in the Treatment of Shoulder Pain: The Adhesive Capsulitis Embolization Study. *Journal of Vascular and Interventional Radiology*. 2022;33(2):177-182. doi:10.1016/j.jvir.2021.10.031

14.	Okuno Y, Yasumoto T, Koganemaru M, et al. Transarterial Embolization of Neovascularity for Refractory Nighttime Shoulder Pain: A Multicenter, Open-Label, Feasibility Trial. *Journal of Vascular and Interventional Radiology*. 2022;33(12):1468-1475.e8. doi:10.1016/j.jvir.2022.08.016
